# Diameter, height and species of 42 million trees in three European landscapes generated from field data and airborne laser scanning data

**DOI:** 10.12688/openreseurope.15373.1

**Published:** 2023-02-14

**Authors:** Raphaël Aussenac, Jean-Matthieu Monnet, Matija Klopčič, Paweł Hawryło, Jarosław Socha, Mats Mahnken, Martin Gutsch, Thomas Cordonnier, Patrick Vallet

**Affiliations:** 1 Université Grenoble Alpes, INRAE, LESSEM, 2 rue de la Papeterie-BP 76, F-38402 St-Martin-d'Hères, France; 2 Forêts et Sociétés, Université de Montpellier, CIRAD, Montpellier, France; 3 CIRAD, UPR Forêts et Sociétés, Yamoussoukro, Cote d'Ivoire; 4 University of Ljubljana, Biotechnical Faculty, Department of Forestry and Renewable Forest Resources, Jamnikarjeva 101, 1000 Ljubljana, Slovenia; 5 Department of Forest Resources Management, Faculty of Forestry, University of Agriculture in Krakow, Al. 29 Listopada 46, 31-425 Krakow, Poland; 6 Potsdam Institute for Climate Impact Research (PIK), Member of the Leibniz Association, Telegrafenberg, 14473 Potsdam, Germany; 7 Office National des Forêts, Département Recherche Développement Innovation, Direction Territoriale Bourgogne-Franche-Comté, 21 rue du Muguet, 39100 Dole, France

**Keywords:** forest, inventory, landscape, tree-level, airborne laser scanning, downscaling

## Abstract

Ecology and forestry sciences are using an increasing amount of data to address a wide variety of technical and research questions at the local, continental and global scales. However, one type of data remains rare: fine-grain descriptions of large landscapes. Yet, this type of data could help address the scaling issues in ecology and could prove useful for testing forest management strategies and accurately predicting the dynamics of ecosystem services. Here we present three datasets describing three large European landscapes in France, Poland and Slovenia down to the tree level. Tree diameter, height and species data were generated combining field data, vegetation maps and airborne laser scanning (ALS) data. Together, these landscapes cover more than 100 000 ha and consist of more than 42 million trees of 51 different species. Alongside the data, we provide here a simple method to produce high-resolution descriptions of large landscapes using increasingly available data: inventory and ALS data. We evaluated the overall reliability of our workflow by comparing the stands dominant heights measured by ALS to those calculated from the trees we generated. Overall, the landscapes we generated are in good agreement with the landscapes they aim to reproduce.

## Introduction

In recent years, a considerable effort has been made to make forest inventory data available, and to aggregate them at the continent [ Mauri *et al.*, 2017] or at the global scale [ Cazzolla Gatti *et al.*, 2022; Liang *et al.*, 2016]. These data make it possible to study ecological processes at fine scales (at the inventory plot scale) as well as at coarse scales (by aggregating inventory plots). At the forest or landscape scale however, they are of limited use as they hardly capture forest- or landscape-level ecological processes. Denser networks of inventory plots or large-scale inventories are needed. However, beyond a certain area, large-scale inventories become too costly and plot networks are preferred. Yet, fine-grain descriptions of large forest areas could help better understand at which spatial scale ecological processes have an effect and thus help address the scaling issues in ecology [ With, 2019]. Such data could also prove useful for testing forest management strategies and accurately predicting the evolution of ecosystem services.

Airborne Laser Scanning (ALS) surveys are a promising way forward to address this challenge, as they can provide high-resolution data over wide areas. However, retrieving individual tree attributes from ALS point clouds remains a challenge in particular in closed-canopy forests. At present, one solution is to combine ALS data with tree-level field data [ Lamb *et al.*, 2018; Silva *et al.*, 2016].

Here we present three datasets describing three large European landscapes in France (Bauges Geopark ≈ 89,000 ha), Poland (Milicz forest district ≈ 21,000 ha) and Slovenia (Snežnik forest ≈ 4700 ha) down to the tree level. Individual trees were generated combining inventory plot data, vegetation maps and ALS data. Together, these landscapes (hereafter virtual landscapes) cover more than 100,000 ha including about 64,000 ha of forest and consist of more than 42 million trees of 51 different species.

In addition to the datasets, we provide here a simple method to predict the diameter, height and species of all trees in a landscape using increasingly available data: inventory and ALS data. This method also has the advantage of being fast: less than 5 hours on a six-core laptop are needed to generate the 35 million trees making up the 51,500 ha of forest in the Bauges Geopark.

## Methods

### Three study areas

**Figure 1.**
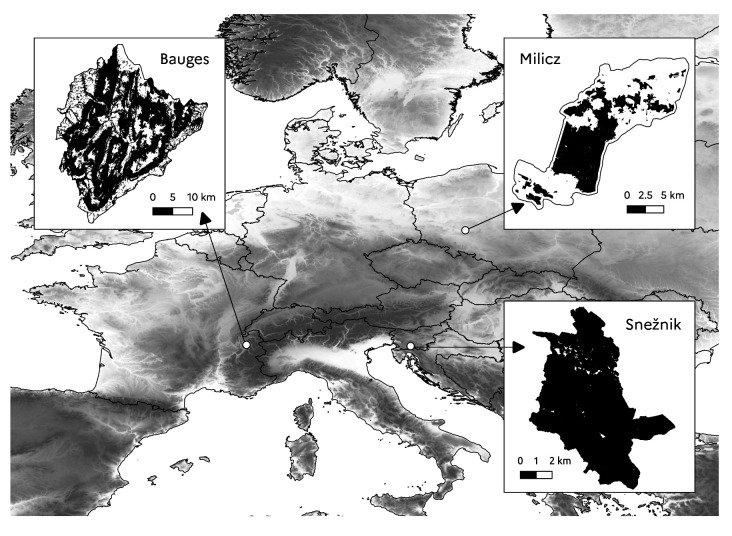
Location of study areas. The black areas show the forested areas.

Three European study areas were used as bases for our virtual landscapes: the Bauges Geopark, the Milicz forest district and the Snežnik forest ( Figure 1).

The Bauges Geopark is a mountainous area located in the French Alps between 255 and 2672 m above sea level (a.s.l.). It is a karst mountain range characterised by a steep and irregular topography. The annual rainfall is about 1100 mm, and the average annual temperature is 8°C at Bellecombe-en-Bauges (850 m a.s.l.). Monthly temperatures range from -1.3 to 17.1°C. The Bauges Geopark covers a total area of 89,324 ha including 51,564 ha of forest (21,073 ha of public forest and 30,491 ha of private forest). The main tree species are beech ( *Fagus sylvatica*), fir ( *Abies alba*) and spruce ( *Picea abies*) which are mostly found in uneven-aged mixed stands, but the area is characterised by a great diversity of tree species. In particular, mixed stands of broadleaf species are found at low elevation.

The Milicz forest district is located in the province of Lower Silesia in south west Poland at a mean elevation of 126 m a.s.l. (elevation ranging from 96 to 227 m a.s.l.). Much of the area is almost flat or slightly undulating with gentle slopes. This part of the landscape is covered by developed terraces and aeolian formations. The remaining part of the landscape is a slightly undulating moraine plateau above which irregularly shaped moraine hills are found. The average annual rainfall is 565 mm and the mean annual temperature is 8.2°C. Monthly temperatures range from -1.3 to 17.8°C. The Milicz forest district covers a total area of 21,086 ha including 7713 ha of public forest. Small patches of private forest are also found in the landscape but they were not considered here as no field data were collected there. The public forest is largely dominated by pure stands of Scots pine ( *Pinus sylvestris*). Pure and mixed stands of oak ( *Quercus robur*) and beech are also found, but in a much smaller proportion.

The Snežnik forest is located in the Dinaric Mountains in southern Slovenia between 572 and 1792 m a.s.l. The Dinaric Mountains are a karst mountain range composed mainly of limestone and dolomite and characterised by an irregular and diverse topography and rockiness. The area has abundant precipitation (over 2000 mm annually on average), which is evenly distributed throughout the year. The average annual temperature is 6.5°C, with a mean monthly maximum temperature of around 16°C in July and a mean minimum of -3.4°C in January. The study area spans over 4725 ha and is almost completely covered by public forest (4660 ha). The main tree species are fir and beech, which are mostly found in uneven-aged mixed stands. Interestingly, in this study area, the upper forest limit is formed by beech stands and not conifer stands.

### General approach

**Figure 2.**
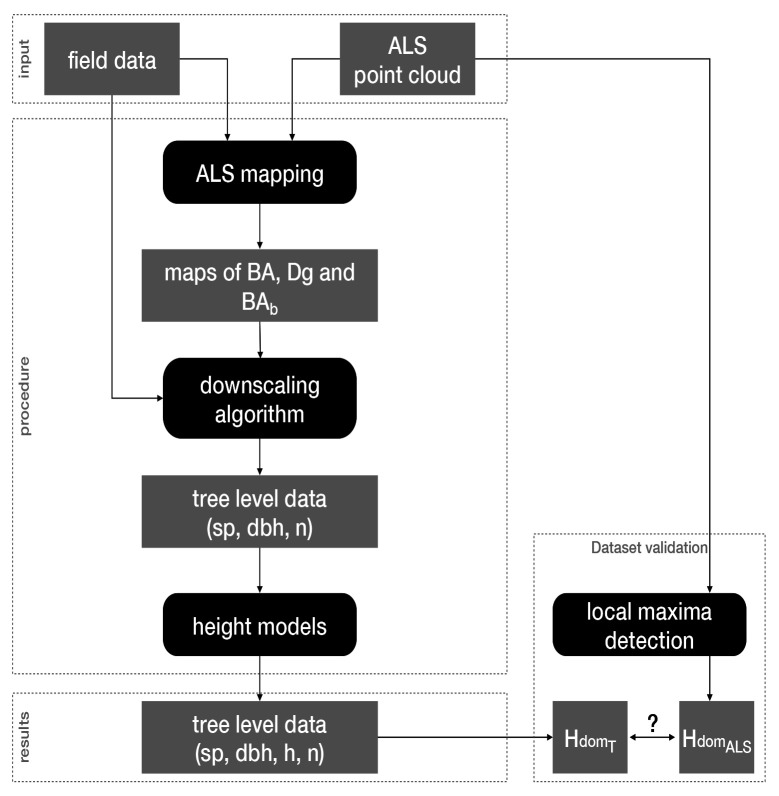
Workflow overview. Black boxes correspond to data generation steps feeding each other with datasets represented by grey boxes. BA: basal area; Dg: mean quadratic diameter; BA _b_: BA proportion of broadleaf trees; sp: species; dbh: diameter at breast height; h: height; n: number of trees; Hdom _ALS_ and Hdom _T_: stands dominant heights measured by ALS or calculated from the generated trees, respectively.

Here we outline the approach we adopted to produce the virtual landscapes corresponding to our three study areas ( Figure 2).

First, we produced raster maps of stand total basal area (BA), mean quadratic diameter (Dg) and proportion of broadleaf trees BA (BA _b_) at a 25 m resolution (see *ALS mapping*). For that, we used ALS point clouds along with field data (tree diameter and species identity). Thereafter, we generated trees in each 25x25 m ^2^ cell, specifying their diameter at breast height (dbh), number (n) and species (sp; see *Downscaling algorithm*). For that, we first assigned to each cell a stand from the field data based on the similarity of their BA, Dg and BA _b_ values. We then transformed the structure of the stand chosen from the field data (by changing the trees dbh, basal area and weight) to reach the BA and BA _b_ values of the cell. Finally, we used diameter-height models to assign heights (h) to all trees (see *Heights models*).

We evaluated the overall reliability of our workflow, *i.e.* its ability to produce virtual landscapes as close as possible to the real ones, by comparing the stands dominant heights measured by ALS (Hdom _ALS_) to those calculated from the trees we generated (Hdom _T_; see *Dataset validation*).

### ALS mapping

The so-called ”area-based” method is a workflow commonly implemented for mapping stands variables in operational conditions [ White *et al.*, 2013]. It is based on the synergistic use of field plots and ALS point clouds. Estimation models for target forest variables are fitted with point clouds statistics, also called metrics, as predictor variables. Field plots are used for training the models. For the mapping step the predictor variables are computed in each cell of a raster layout over the whole acquisition area, and then the models are applied to obtain wall-to wall-estimates. This workflow was implemented in each study area.

***Forest areas.*** Reference areas for forest mapping were defined as the intersection of two layers for each site, one defining the administrative boundary and one defining the forest mask. Those extents are respectively:

- Bauges: the Geopark administrative extent with the forest mask defined by the BD Forêt v2 from the National Institute of Geographic and Forest Information [ IGN, 2019], excluding the ”herbaceous”, ”moors” and ” *Populus* plantations” categories;
- Milicz: the public forests of Milicz with the forest mask defined by the Forest Data Bank [ Bureau for Forest Management and Geodesy, 2020];
- Snežnik: the forest management units of Leskova Dolina and Snežnik with the forest mask defined by Snežnik-forest cover [ Service, 2020].

***Field data.*** In the Bauges, a local forest inventory with 320 plots was implemented in 2018. On each plot, all living trees with a dbh larger than 17.5 cm and within a 15 m horizontal distance from the plot centre had their dbh, position and species recorded. Trees with a dbh between 7.5 and 17.5 cm were counted according to simplified categories of diameter and species (coniferous / broadleaf). Plot centres were geolocated with survey-grade GNSS (Global Navigation Satellite System) receivers. Plots co-registration with the ALS data was improved when possible by comparing the positions of trees with the Canopy Height Model (CHM) derived from the point cloud.

At Milicz, a local forest inventory with 901 plots of 12.62 m radius was carried out in 2015. Species and diameter of all living trees with dbh above 7 cm were recorded. Plot centres were geolocated with survey-grade GNSS receivers.

At Snežnik, a total of 515 plots were inventoried, in 2013 for plots located in the Leskova Dolina management unit and in 2014 for plots located in the Snežnik management unit. Trees with a dbh above 30 cm within a 12.61 m distance from the plot centre had their diameter and species recorded. Trees with a dbh between 10 and 30 cm were recorded within a 7.98 m distance from the plot centre. Plot centres were geolocated with commercial-grade GNSS receivers.

The following stand-level variables were computed for each plot: total basal area (BA) in m ^2^.ha ^-1^, mean quadratic diameter (Dg) in cm and the proportion of broadleaf species in basal area (BA _b_). Weights were applied to correct for sampling intensity in the case of nested plots (Bauges and Snežnik).

***ALS data.*** The Bauges was covered by two ALS acquisitions with different settings and equipment. The southern part was covered between June and September 2016, the northern part in September 2018. Mean point densities were respectively 5.5 and 24.4 m ^-2^. Intensity values were normalised by dataset, by subtracting the mean and dividing by the standard deviation of intensity values of points located inside the extent of field plots covered by each acquisition.

Milicz was covered by an ALS acquisition in August 2015. The average point density was 16.8 m ^-2^. The point cloud contains colour values extracted from aerial pictures with near infra-red, red and green bands. Snežnik was covered by an ALS acquisition between February 14th and November 21st 2014. Forests might have been both in leaf-on and leaf-off conditions. The average point density was 18.1 m ^-2^. An ice storm occurred in Leskova Dolina management unit between January 30th and February 10th 2014. This event damaged the forest stands, and happened between the field inventory and the ALS acquisition. It affected the quality of the derived maps (see *Mapping*) and the realism of our virtual landscape (see *Dataset validation*).

***ALS metrics.*** All computations were performed with R software. Terrain metrics (aspect, elevation and slope) were computed by fitting a plane surface to points classified as ground. Before the computation of vegetation metrics, ALS point clouds were normalised, *i.e.* height above ground was computed for each point. Two types of metrics were then computed from the points classified as vegetation with a height above 2 meters (this limit was set to remove points of shrubs and low vegetation from the analysis):

- Point cloud metrics were directly computed from the point cloud or from the derived CHM, using the aba metrics function from the lidaRtRee R package. Those metrics summarise the geometry of the point cloud in a given area.
- Tree metrics were computed with the std tree metrics function from the characteristics of local maxima extracted tree_segmentation function) from the CHM. CHM resolution was set to 0.5 m at Milicz, and 1 m at Snežnik and the Bauges due to higher variability of point density. Local maxima with a height lower than 5 m were discarded. Those metrics summarise the characteristics of trees detected in a given area of the point cloud. One of the tree metrics is the ALS dominant height (Hdom _ALS_), which is the mean height of the six highest local maxima. In case less than six maxima were present, the mean height of all maxima was used.

The metrics were computed for each field plot based on the point cloud located inside their extent, in order to build the dataset for model calibration (training step). The metrics were also computed in each 25x25 m ^2^ cell of the raster layout covering each acquisition, in order to build the prediction dataset (mapping step).

***Models.*** For BA and Dg, we searched for the linear regression model that yielded the highest adjusted-R ^2^ with at most *n* = 6 independent variables among the above-mentioned ALS metrics. The model was given by:


$$\;\hat{y}=a_{0}+\sum_{i=1}^{n}a_{i}x_{i}\;(1)$$

with $\hat{y}$ the estimated value, ( *a _i_*) *i*∈{0,..., *n*} the model parameters and ( *x _i_*) *i*∈{1,..., *n*} the selected metrics. Two data transformations were also tested: a logarithm transformation of all variables and a Box-Cox transformation of the dependent variable. The logarithm transformation of all variables turns the model at Equation 1 into:


$$\;\hat{y}=e^{\left(a_{0}\right)}\times \prod_{i=1}^{n}x_{i}^{a_{i}}\;(2)$$

A bias correction factor had to be applied to the fitted values to obtain the predictions ( *P*):


$$\;P=\hat{y}\times e^{\left(\frac{v}{2}\right)}\;(3)$$

with *υ* the variance of the model residuals.

The Box-Cox transformation consists in determining the *λ* parameter that best normalises the distribution of the dependent variable ( *Y* ). It is determined using the maximum likelihood-like approach of Box & Cox [1964]( *powerTransform* function of car R package). *Y* is given by:


$$\;Y=\frac{\left(y^{\lambda }-1\right)}{\lambda }\;(4)$$

Equation 1 is then fitted with *Y* instead of *y*. The predictions *P* are obtained by applying the inverse Box-Cox transformation to the fitted values $\hat{Y}$ and a bias correction factor:


$$\;P={\left(\lambda \hat{Y}+1\right)}^{\frac{1}{\lambda }}\times \left(1+\frac{v}{2}\times \frac{1-\lambda }{(\lambda \hat{Y}+1{)}^{2}}\right)\;(5)$$

For broadleaf proportion (BA _b_), values are bounded to [0, 1]. A binomial generalised linear model with logit link was therefore fitted with the *glm R* function. The model was given by:


$$\;log\left(\frac{\hat{BA_{b}}}{1-\hat{BA_{b}}}\right)=a_{0}+\sum a_{i}x_{i}\;(6)$$

All metrics were at first included in the model and then a stepwise selection was used to reduce their number ( *stepAIC* function of the *MASS R* package).

***Stratification.*** When calibrating a statistical relationship between forest stand variables, which are usually derived from diameter measurements and ALS metrics, one relies on the hypothesis that the interaction of laser pulses with the leaves and branches structure is constant on the whole area. However, differences can be expected either due to variations in acquisition settings (flight parameters, scanner model), in forests (stand structure and composition) or in topography (slope). Better models might be obtained when calibrating stratum-specific relationships, provided each stratum is more homogeneous regarding the laser interaction with the vegetation. A trade-off has to be achieved between the within-strata homogeneity and the number of available plots for calibration in each stratum.

Depending on the study areas, different ancillary data are available for stratification. At the Bauges, two layers were used: species composition (mixed, broadleaf, coniferous) derived from the BD Forêt v2 and ALS survey. At Milicz, the following information was available for a total of 2175 stands: dominant species (coniferous, *Quercus*, other broadleaf) and stand age. At Snežnik, the following information was available for a total of 1536 stands: forest management unit (FMU: Snežnik or Leskova Dolina) and broadleaf proportion in volume, which is converted into a two (broadleaf or coniferous) or three-levels factor (adding the mixed category).

Field plots and raster cells were assigned to the category of the polygon which contains their centres.

***Mapping.*** Stratifications were compared based on expert knowledge taking into account the following criteria: minimum number of observations in strata, prediction error and number of variables in the model. The retained stratifications for the prediction models and the root mean square error (RMSE) of prediction estimated in leave-one-out cross validation are presented in Table 1.

**Table 1.** Stratification and root mean square error (RMSE) of predictions for the three study areas and three forest variables. BA: basal area (m ^2^.ha ^-1^); Dg: mean quadratic diameter (cm); BA _b_: broadleaf BA proportion.

study area	Variable	RMSE	Stratification: number and combinations
Bauges	BA	8.3	6: composition x ALS survey
	Dg	4.2	6: composition x ALS survey
	BA _b_	20.3	3: composition
Milicz	BA	5.4	7: (coniferous x 5 age classes), *Quercus sp*., other broadleaf
	Dg	3.7	3: coniferous, *Quercus sp*., other broadleaf
	BA _b_	12.9	2: coniferous, broadleaf
Snežnik	BA	9.6	4: FMU x composition (2 classes)
	Dg	7.6	6: FMU x composition (3 classes)
	BA _b_	19.3	2: FMU

**Figure 3.**
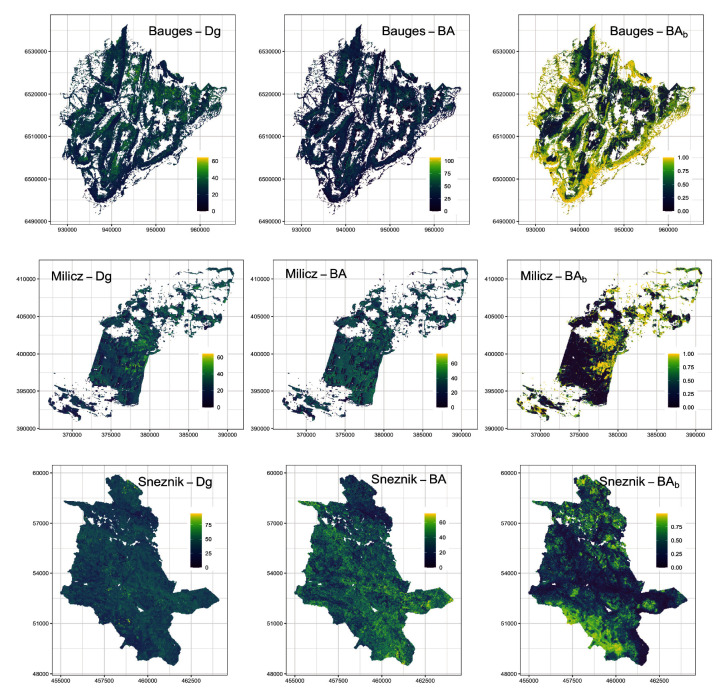
Airborne laser scanning (ALS) maps of forest variables for our three study areas at a 25 m resolution. Dg: mean quadratic diameter (cm), BA: basal area (m ^2^.ha ^-1^) and (BA _b_): proportion of broadleaf BA.

Prediction accuracy is better for mean diameter and lower for BA, which is common when estimated with ALS. Precision is quite low for broadleaf proportion, which could be expected as spectral data are usually better than ALS at classifying species. Prediction accuracy was higher at Milicz, intermediate at the Bauges and lower at Snežnik. Milicz was well suited for making predictions with its dense ALS data, homogeneous stands and precise co-registration. The Bauges has precise co-registration, but heterogeneous forest stands and two different ALS datasets. At Snežnik the data were much noisier, especially because of the ice storm event. The maps we created are presented in Figure 3.

### Downscaling algorithm

***Field data.*** At Milicz and Snežnik, we used the same dbh measurements as those used to calibrate the ALS models (from 901 plots at Milicz and from 515 plots at Snežnik, see *ALS mapping - Field data*). At the Bauges, we could not use the dbh measurements used to calibrate the ALS models because trees with a dbh smaller than 17.5 cm were not measured but counted by diameter classes. Instead, we used the tree diameter measurements from the 258 forest plots of the French National Forest Inventory (NFI) located in the study area. Those plots were inventoried between 2005 and 2018. They consist of three concentric plots of 6 m, 9 m and 15 m radius, where small (7.5 *<* dbh *<* 22.5 cm), medium (dbh *<* 37.5 cm) and big trees (dbh *>* 37.5 cm) were measured, respectively. At the Bauges, we used an additional information on forest vegetation: the map of forest types [ IGN, 2019], which we also used to delineate the forest areas (see *Forest areas*).

***Algorithm.*** Our algorithm consisted in associating to each 25×25 m ^2^ cell a field plot based on the similarity of their dendrometrical variables, and then in modifying the trees dbh, basal area and weight of this field plot in order to reach the total BA and the proportion of broadleaf BA (BAb) of the cell ( *i.e.* the values provided by the ALS maps). The algorithm breaks down as follows:

1. First, we calculated the total basal area (BA), mean quadratic diameter (Dg) and proportion of broadleaf BA (BA _b_) of all field plots.
2. Second, we associated to each 25×25 m ^2^ cell a field plot based on the similarity of their BA, Dg and BA _b_.
  (a) For this, we scaled the values of BA, Dg and BA _b_ between 0 and 1. We scaled the ALS and field data together to account for the possible differences in their range.
  (b) We then calculated the Euclidean distance between each cell and each field plot in the three-dimensional space made up by the scaled values of BA, Dg and BA _b_.
  (c) Finally, we associated to each cell the closest field plot in this three-dimensional space. For the Bauges study area, we assigned to each 25×25 m ^2^ cell a forest type ( *e.g.* pure beech, mixed deciduous forest, among others) from the map of forest types. We then associated the closest field plot sharing the same forest type to each cell.
3. Third, we transformed the field plots stand structure so that it matched the BA and BA _b_ values of the cells they were associated with.
  (a) For this, we first calculated *α*, a multiplier correction coefficient to apply to all trees dbh of the field plots. *α* is given by:
$$\;\alpha =\frac{Dg_{ALS}}{Dg_{F}}\;(7)$$
    with *Dg _ALS_* the Dg value of the cell given by the ALS mapping, and *Dg _F_* the Dg value calculated with the dbh of the trees from the field plot.
  (b) Thereafter, we calculated the weight per ha of each tree as follows:
$$\;\omega =\frac{40000}{\pi }\times \frac{ba_{tree_{ALS,F}}}{({\alpha \text{.}dbh}_{F}{)}^{2}}\;(8)$$
    where *dbh _F_* is the tree dbh in the field plot, and *ba _treeALS,F_* is the tree individual basal area derived from the ALS mapping and the field plot data using the following equation:
$$\;ba_{tree_{ALS,F}}=BA_{ALS}\times Prop_{{BC}_{\mathrm{ALS}}}\times Prop_{S_{pF}}\times Prop_{tree_{F}}\;(9)$$
    where *BA _ALS_* is the total BA of the cell given by the ALS mapping, *PropBC _ALS_* is the BA proportion of broadleaf (resp. coniferous) trees given by the ALS mapping, *PropS _pF_* is the BA proportion of species *S _p_* in broadleaf (reps. coniferous) species in the field plot, and *Prop _treeF_* is the BA proportion of this tree in species *S _p_* in the field plot.
  (c) Finally, we divided *ω* by 16 and performed a Bernouilli draw on the decimal part of the obtained values (as an example, a weight of 5.63 has a 63% chance of becoming 6, and a 37% chance of becoming 5) to get integer values corresponding to the weight of the trees in the 25×25 m ^2^ cells. As this rounding of the weights slightly modifies the stand total BA, we transformed again the trees dbh to reach the total BA provided by the ALS mapping using the trees BA and their integer weight ( *ω _int_*) as follows:
$$\;dbh_{final}=\sqrt{\frac{40000}{\pi }\times \frac{\frac{ba_{tree_{ALS,F}}}{16}}{\omega_{int}}}\;(10)$$
    As this last transformation only compensates for the rounding, the changes in dbh are minor.

This procedure has multiple benefits (see proofs in *Extended data*): it makes it possible to reach the BA and BA _b_ values given by the ALS mapping. It also maintains the Dg ratios observed on the field plots between the different species. The Bernouilli draw used to get integer tree weights only adds a minor variability. We created the three virtual landscapes by applying this algorithm to each study area separately.

### Heights models

We developed individual diameter-height models for the three study areas to assign heights to all generated trees.

***Field data.*** At Snežnik and Milicz, the diameter and height measurements come from the same field plots used for the ALS models calibration (see *ALS mapping - Field data*). At the Bauges, no height measurements were collected in the field plots used to calibrate the ALS models. We therefore used the tree diameter and height measurements of the 240 French NFI plots located in the study area (inventoried between 2005 and 2016). At Milicz and the Bauges, the heights were measured for all species in all diameter classes. At Snežnik, tree heights were measured only on two to four trees from the upper layer. The number of trees with both diameter and height measurements in each study area is summarised per species in Table 2.

**Table 2.** Number of trees for the diameter-height models calibration in each study area and for each species. For each study area, all the species with less than 100 observations are grouped into the ”other species” category.

Species	Number of trees for			
	Bauges	Milicz	Snežnik
*Abies alba*	468		638
*Acer pseudoplatanus*	181	228	
*Alnus glutinosa*		823	
*Betula pendula*		1 519	
*Carpinus betulus*		808	
*Fagus sylvatica*	705	2 199	435
*Fraxinus excelsior*	209		
*Larix decidua*		709	
*Picea abies*	551	2 183	325
*Pinus sylvestris*		24 995	
*Prunus serotina*		191	
*Quercus petraea*	130		
*Quercus rubra*		308	
*Quercus* undefined *		1 916	
*Tilia cordata*		311	
Other species	642	522	29
TOTAL	2 886	36 712	1 427

*At Milicz, the *Quercus* undefined is mainly *Quercus robur*.

***Models.*** We used a mixed effect model to predict individual tree height from the ratio between the tree dbh and the stand Dg (to account for the tree social status) and from the stand Dg (to account for the stand development stage). We considered the site effect as a random effect. Finally, as the variance of height increases with height due both to increasing measurement errors and to individual cumulative variations, we accounted for heteroscedasticity by modelling the error term with a power of the fitted values. The model is given by:


$$h_{tot}=1.3+(1+\alpha_{site})\times \alpha_{sp}\times \left(1-e^{\left(-\alpha_{1}\times Dg^{\alpha_{2}}\right)}\right)\times {\left(1-e^{\left(-\beta_{sp}\times \frac{dbh}{Dg}\right)}\right)}^{\gamma }+\;(11)$$

where *α _sp_*, *α*_1_, *α*_2_, *β _sp_* and *γ* are parameters to be estimated; and *α _site_*, a random effect accounting for the site effect. This model has an asymptotic form: *α _sp_* corresponds to the species-specific asymptotic value, and *β _sp_* is the species-specific speed for reaching the asymptotic value.

At Snežnik, most of the trees selected for height measurement were dominant or co-dominant trees. Moreover, more than half of the plots only had two observations. This precludes to fit the part of the curve with small diameters within the stand. We solved this issue by assuming that the within-stand relationship at the Bauges was similar at Snežnik, as these landscapes are quite similar in terms of species, stand structure (mostly uneven-aged), or elevation (mountains). Therefore, for Snežnik height predictions, we used the *β _sp_* and *γ* fitted values of the Bauges model.

We fitted one mixed effect model for each study area using the *nlme* function from the *nlme R* package. We modelled the residual errors using a *varPower* function of the fitted values. The parameters are presented in Table 3, Table 4, and Table 5 for the three study areas.

**Table 3.** Parameters of the Bauges diameter-height model.

Parameter	Value	Standard error	p-value
*α _Fa.sy._*	41.05595	4.3	*<*10 ^–3^
*α _Pi.ab._*	55.11821	5.8	*<*10 ^–3^
*α _Ab.al._*	48.46640	5.1	*<*10 ^–3^
*α _Fr.ex._*	40.94293	4.3	*<*10 ^–3^
*α _Ac.ps._*	37.95001	4.0	*<*10 ^–3^
*α _Qu.pe._*	36.64676	4.2	*<*10 ^–3^
*α _OtherSp._*	36.87834	3.8	*<*10 ^–3^
*α*_1_	0.01594	0.0030	*<*10 ^–3^
*α*_2_	1.26326	0.10	*<*10 ^–3^
*β _Fa.sy._*	1.71474	0.08	*<*10 ^–3^
*β _Pi.ab._*	0.99226	0.05	*<*10 ^–3^
*β _Ab.al._*	1.17894	0.06	*<*10 ^–3^
*β _Fr.ex._*	2.01951	0.12	*<*10 ^–3^
*β _Ac.ps._*	2.08068	0.12	*<*10 ^–3^
*β _Qu.pe._*	1.56216	0.16	*<*10 ^–3^
*β _OtherSp._*	1.84067	0.08	*<*10 ^–3^
*γ*	1.42595	0.05	*<*10 ^–3^
Power of the variance model			0.51
Standard deviation of the plot level random effect			0.14
Standard deviation of residual error			0.59

**Table 4.** Parameters of the Milicz diameter-height model.

Parameter	Value	Standard error	p-value
*α _Pi.sy._*	48.55802	2.3	*<*10 ^–3^
*α _Fa.sy._*	48.01692	2.3	*<*10 ^–3^
*α _P i.ab._*	60.35196	3.1	*<*10 ^–3^
*α _Qu.un._*	52.24210	2.5	*<*10 ^–3^
*α _Be.pe._*	51.60844	2.5	*<*10 ^–3^
*α _Al.gl._*	49.34039	2.4	*<*10 ^–3^
*α _Ca.be._*	36.73985	1.8	*<*10 ^–3^
*α _La.de._*	52.06992	2.5	*<*10 ^–3^
*α _Ti.co._*	45.25535	2.4	*<*10 ^–3^
*α _Qu.ru._*	45.74754	2.4	*<*10 ^–3^
*α _Ac.ps._*	41.50894	2.2	*<*10 ^–3^
*α _Pr.se._*	36.18532	2.9	*<*10 ^–3^
*α _OtherSp._*	54.94652	2.8	*<*10 ^–3^
*α*_1_	0.01958	0.001	*<*10 ^–3^
*α*_2_	1.13831	0.035	*<*10 ^–3^
*β _Pi.sy._*	2.73192	0.024	*<*10 ^–3^
*β _Fa.sy._*	1.98085	0.032	*<*10 ^–3^
*β _Pi.ab._*	1.20700	0.035	*<*10 ^–3^
*β _Qu.un._*	1.62943	0.027	*<*10 ^–3^
*β _Be.pe._*	2.11097	0.037	*<*10 ^–3^
*β _Al.gl._*	2.04760	0.045	*<*10 ^–3^
*β _Ca.be._*	2.86677	0.063	*<*10 ^–3^
*β _La.de._*	2.33369	0.050	*<*10 ^–3^
*β _Ti.co._*	1.89682	0.064	*<*10 ^–3^
*β _Qu.ru._*	2.38748	0.095	*<*10 ^–3^
*β _Ac.ps._*	2.56340	0.102	*<*10 ^–3^
*β _Pr.se._*	2.04373	0.150	*<*10 ^–3^
*β _OtherSp._*	1.50792	0.019	*<*10 ^–3^
*γ*	1.55264	0.040	*<*10 ^–3^
Power of the variance model			0.16
Standard deviation of the plot level random effect			0.09
Standard deviation of residual error			1.09

**Table 5.** Parameters of the Snežnik diameter-height model.

Parameter	Value	Standard error	p-value
*α _Ab.al._*	66.17413	5.4	*<*10 ^–3^
*α _Fa.sy._*	53.81402	4.4	*<*10 ^–3^
*α _Pi.ab._*	76.82544	6.3	*<*10 ^–3^
*α*_1_	0.0251	0.0036	*<*10 ^–3^
*α*_2_	1.00672	0.075	*<*10 ^–3^
*β _Ab.al._* *	1.17894	* taken from the Bauges model	
*β _Fa.sy._* *	1.71474		
*β _Pi.ab._* *	0.99226		
γ*	1.42595		
Power of the variance model			-0.56
Standard deviation of the plot level random effect			0.077
Standard deviation of residual error			15.8

## Dataset validation

### Method

To assess the realism of the virtual landscapes we generated, we compared the stand dominant heights estimated by ALS (Hdom _ALS_) to those calculated from the trees we generated (Hdom _T_). We expect Hdom _ALS_ to be as close to reality as possible, as tree height is among the most reliable ALS measurement [ Van Leeuwen & Nieuwenhuis, 2010] and can be derived from ALS data with little processing and no field data. Hdom _ALS_ therefore serves here as a reference to which Hdom _T_ is compared. As shown in Figure 2 Hdom _ALS_ is totally independent from the procedure that generates the trees. Thus, comparing Hdom _ALS_ and Hdom _T_ makes it possible to evaluate the overall reliability of our workflow.

In practice, Hdom _T_ is calculated as the mean height of the six highest trees, while Hdom _ALS_ is calculated as the mean height of the six highest local maxima (see *ALS metrics*). In case less than six trees/maxima were found, the mean height of all trees/maxima was used. These dominant heights are calculated at the 25×25 m ^2^ cell level.

### Results

**Figure 4.**
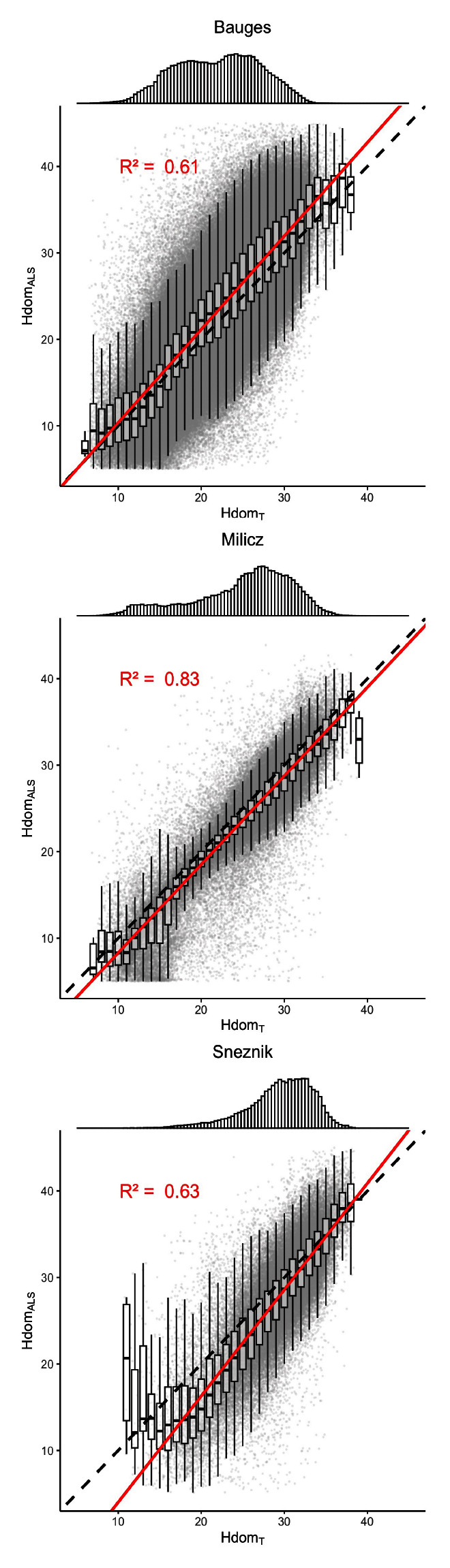
Comparison of the stands dominant heights measured by ALS (Hdom _ALS_) to those calculated from the generated trees (Hdom _T_). The top panels show the distribution of Hdom _T_. The dashed lines indicate the *y* = *x* line. The red lines correspond to the regression lines. The regression R-Squared values are shown in red.

Overall, with R ^2^ values ranging from 0.61 to 0.83 ( Figure 4), Hdom _ALS_ and Hdom _T_ were consistent with each other. This indicates that the virtual landscapes are in good agreement with the landscapes they aim to reproduce. However, Hdom _ALS_ and Hdom _T_ showed some divergence at Snežnik: Hdom _T_ tends to be overestimated as Hdom _ALS_ decreases. This could be due to the ice storm that occurred between the field inventory and the ALS acquisition and that might have biased the ALS models.

## Virtual landscapes overview

**Figure 5.**
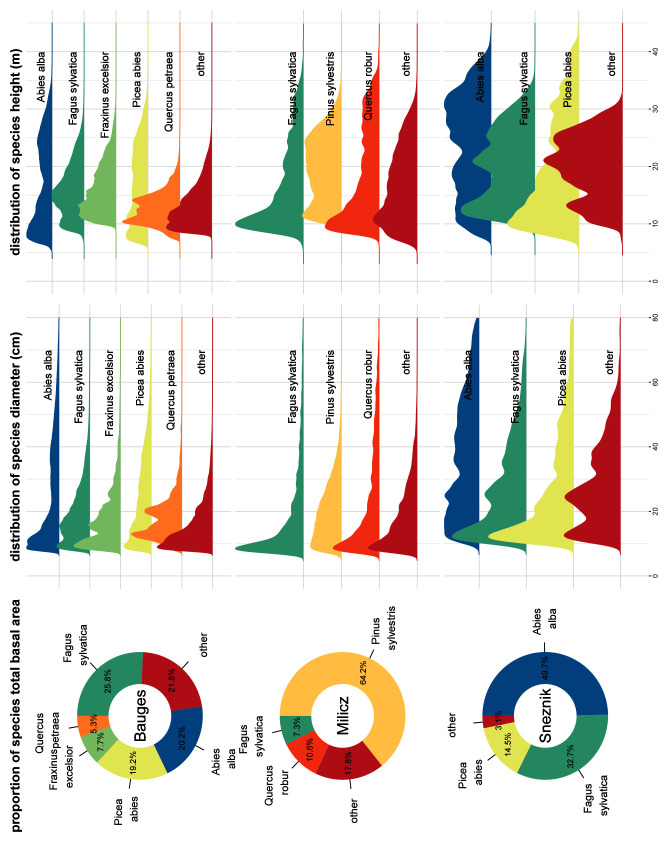
Main species basal area proportion, diameter distribution and height distribution in the three virtual landscapes. Species accounting for less than 5% of the virtual landscapes total basal area were grouped in the ’other’ category.

Overall, 42,394,479 trees belonging to 51 different species were generated: 35,134,985 trees of 40 different species were generated at the Bauges, 5,726,420 trees of 32 different species at Milicz and 1,533,074 trees of 16 different species at Snežnik. The main species BA proportion as well as their h and dbh distributions are shown in Figure 5 for each virtual landscape.

## Data Availability

**Bauges** The maps of forest types (BD Forêt®V2) are available to download from the National Institute for Geographic and Forestry Information website at https://geoservices.ign.fr/bdforet, under the Etalab open license 2.0. The French National Forest Inventory data are available to download from the National Institute for Geographic and Forestry Information website at https://inventaire-forestier.ign.fr/dataifn/, under the Etalab open license 2.0. The local forest inventory dataset is available for non-commercial use upon request to Jean-Matthieu Monnet ( jean-matthieu.monnet@inrae.fr). A data sharing agreement will have to be established, with the following restrictions: data are available for internal use only and cannot be distributed; results obtained from the data can be displayed or distributed provided they do not allow the estimation of growing stock in individual private properties; data funding (Ademe grant 1703C0069) should be cited. ALS data in the northern part (Haute-Savoie) are available to download from the Recherche Data Gouv dataverse at https://doi.org/10.57745/ZUT1MJ, under the Etalab open license 2. ALS data in the southern part (Savoie) can be purchased upon request to (Régie de Gestion des Données Savoie Mont Blanc) at https://www.rgd.fr/. **Milicz** The stand data in the ESRI Shapefile format are available to download from the Polish Forest Data Bank at https://www.bdl.lasy.gov.pl/portal/wniosek-en. The local forest inventory dataset and ALS data are available for non-commercial use upon request to Jarosław Socha ( jaroslaw.socha@urk.edu.pl). A data sharing agreement will have to be established, with the following restrictions: data are available for internal use only and cannot be distributed; data funding (REMBIOFOR - BIOSTRATEG1/267755/4/NCBR/2015) should be cited. **Sneznik** The forest inventory data (in *.xlsx and *.shp formats) and maps of forest types and species mixture (in *.shp format) are available upon request to Slovenia Forest Service ( zgs.tajnistvo@zgs.si; rok.pisek@zgs.si). A data sharing agreement will have to be established, with the following restrictions: data are only available for the study that is the subject of the agreement; Slovenia Forest Service should be acknowledged for providing the data in all publications. ALS data are available to download from the Slovenian Environment Agency website at http://gis.arso.gov.si/evode, under the terms of the international Creative Commons 4.0 license ( http://www.evode.gov.si/fileadmin/user_upload/Lidar_pogoji_uporabe.pdf): the data user must indicate the data source at each publication of data or products, specifying ”Slovenian Environmental Agency, type of data and period to which the data refer or the date of the database”. Zenodo: I-MAESTRO data: 42 million trees from three large European landscapes in France, Poland and Slovenia. https://doi.org/10.5281/zenodo.7462440 [ Aussenac *et al.*, 2022]. For each virtual landscape we provide a table (in .csv format) with the following columns: cellID25: the unique ID of each 25x25 m ^2^ cell sp: species latin names n: number of trees dbh: tree diameter at breast height (cm) h: tree height (m) We also provide, for each virtual landscape, a raster (in .asc format) with the cell IDs (cellID25) which makes data spatialisation possible. Finally, we provide a proof of how, in the downscaling algorithm, multiplying the trees dbh by the *α* correction coefficient makes it possible to reach the cells BA value derived from the ALS mapping.
