# Diameter, height and species of 42 million trees in three European landscapes generated from field data and airborne laser scanning data

**DOI:** 10.12688/openreseurope.15373.2

**Published:** 2023-12-05

**Authors:** Raphaël Aussenac, Jean-Matthieu Monnet, Matija Klopčič, Paweł Hawryło, Jarosław Socha, Mats Mahnken, Martin Gutsch, Thomas Cordonnier, Patrick Vallet

**Affiliations:** 1 Université Grenoble Alpes, INRAE, LESSEM, 2 rue de la Papeterie-BP 76, F-38402 St-Martin-d'Hères, France; 2 Forêts et Sociétés, Université de Montpellier, CIRAD, Montpellier, France; 3 CIRAD, UPR Forêts et Sociétés, Yamoussoukro, Cote d'Ivoire; 4 University of Ljubljana, Biotechnical Faculty, Department of Forestry and Renewable Forest Resources, Jamnikarjeva 101, 1000 Ljubljana, Slovenia; 5 Department of Forest Resources Management, Faculty of Forestry, University of Agriculture in Krakow, Al. 29 Listopada 46, 31-425 Krakow, Poland; 6 Potsdam Institute for Climate Impact Research (PIK), Member of the Leibniz Association, Telegrafenberg, 14473 Potsdam, Germany; 7 Office National des Forêts, Département Recherche Développement Innovation, Direction Territoriale Bourgogne-Franche-Comté, 21 rue du Muguet, 39100 Dole, France

**Keywords:** forest, inventory, landscape, tree-level, airborne laser scanning, downscaling

## Abstract

Ecology and forestry sciences are using an increasing amount of data to address a wide variety of technical and research questions at the local, continental and global scales. However, one type of data remains rare: fine-grain descriptions of large landscapes. Yet, this type of data could help address the scaling issues in ecology and could prove useful for testing forest management strategies and accurately predicting the dynamics of ecosystem services.

Here we present three datasets describing three large European landscapes in France, Poland and Slovenia down to the tree level. Tree diameter, height and species data were generated combining field data, vegetation maps and airborne laser scanning (ALS) data following an area-based approach. Together, these landscapes cover more than 100 000 ha and consist of more than 42 million trees of 51 different species.

Alongside the data, we provide here a simple method to produce high-resolution descriptions of large landscapes using increasingly available data: inventory and ALS data.

We carried out an in-depth evaluation of our workflow including, among other analyses, a leave-one-out cross validation. Overall, the landscapes we generated are in good agreement with the landscapes they aim to reproduce. In the most favourable conditions, the root mean square error (RMSE) of stand basal area (BA) and mean quadratic diameter (Dg) predictions were respectively 5.4 m ^2^.ha ^-1^ and 3.9 cm, and the generated main species corresponded to the observed main species in 76.2% of cases.

## Introduction

In recent years, a considerable effort has been made to make forest inventory data available, and to aggregate them at the continent [ Mauri *et al.*, 2017] or at the global scale [ Cazzolla Gatti *et al.*, 2022; Liang *et al.*, 2016]. These data make it possible to study ecological processes at fine scales (at the inventory plot scale) as well as at coarse scales (by aggregating inventory plots). At the forest or landscape scale however, they are of limited use as they hardly capture forest- or landscape-level ecological processes. Denser networks of inventory plots or large-scale inventories are needed. However, beyond a certain area, large-scale inventories become too costly and plot networks are preferred. Yet, fine-grain descriptions of large forest areas could help address the pervasive scaling issues in forest ecology, modelling and management. In practice, such data could help better understand at which spatial scale ecological processes emerge in forest ecosystems [ Craven *et al.*, 2020; With, 2019]. They could also be extremely valuable to compare forest dynamics models operating at different scales (organ, tree, stand, landscapes) and evaluate their validity across scales [ Papaik *et al.*, 2010]. They could ultimately help develop and test management strategies at different spatial scales [ Seidl *et al.*, 2013].

Airborne Laser Scanning (ALS) surveys are a promising way forward to address this challenge, as they can provide high-resolution data over wide areas. However, retrieving individual tree attributes from ALS point clouds remains a challenge in particular in closed-canopy forests. At present, one solution is to combine ALS data with tree-level field data [ Lamb *et al.*, 2018; Silva *et al.*, 2016].

Here we present three datasets describing three large European landscapes in France (Bauges Geopark ≈ 89,000 ha), Poland (Milicz forest district ≈ 21,000 ha) and Slovenia (Snežnik forest ≈ 4700 ha) down to the tree level. Individual trees were generated combining inventory plot data, vegetation maps and ALS data. Together, these landscapes (hereafter virtual landscapes) cover more than 100,000 ha including about 64,000 ha of forest and consist of more than 42 million trees of 51 different species.

In addition to the datasets, we provide here a simple method to predict the diameter, height and species of all trees in a landscape using increasingly available data: inventory and ALS data. This method also has the advantage of being fast: about 1 hour on an height-core laptop is needed to generate the 42 million trees making up the 64,000 ha of forest of our three landscapes.

## Methods

### Three study areas

**Figure 1.**
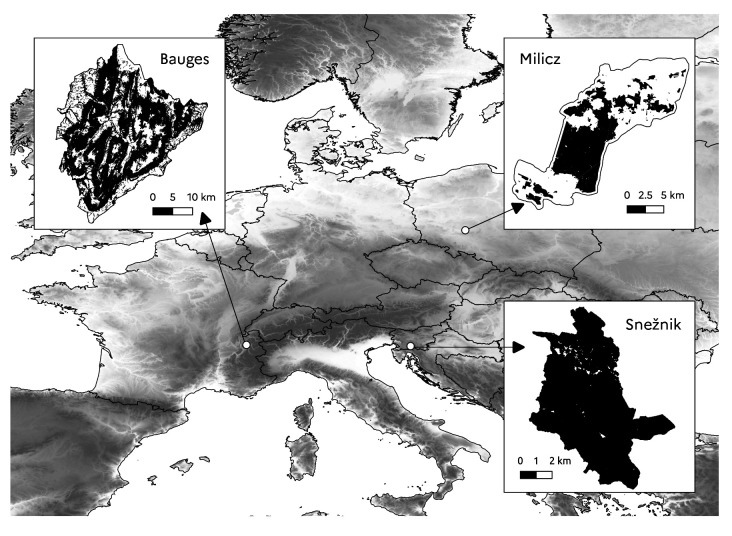
Location of study areas. The black areas show the forested areas.

Three European study areas were used as bases for our virtual landscapes: the Bauges Geopark, the Milicz forest district and the Snežnik forest ( Figure 1).

The Bauges Geopark is a mountainous area located in the French Alps between 255 and 2672 m above sea level (a.s.l.). It is a karst mountain range characterised by a steep and irregular topography. The annual rainfall is about 1100 mm, and the average annual temperature is 8°C at Bellecombe-en-Bauges (850 m a.s.l.). Monthly temperatures range from -1.3 to 17.1°C. The Bauges Geopark covers a total area of 89,324 ha including 51,564 ha of forest (21,073 ha of public forest and 30,491 ha of private forest). The main tree species are beech ( *Fagus sylvatica*), fir ( *Abies alba*) and spruce ( *Picea abies*) which are mostly found in uneven-aged mixed stands, but the area is characterised by a great diversity of tree species. In particular, mixed stands of broadleaf species are found at low elevation.

The Milicz forest district is located in the province of Lower Silesia in south west Poland at a mean elevation of 126 m a.s.l. (elevation ranging from 96 to 227 m a.s.l.). Much of the area is almost flat or slightly undulating with gentle slopes. This part of the landscape is covered by developed terraces and aeolian formations. The remaining part of the landscape is a slightly undulating moraine plateau above which irregularly shaped moraine hills are found. The average annual rainfall is 565 mm and the mean annual temperature is 8.2°C. Monthly temperatures range from -1.3 to 17.8°C. The Milicz forest district covers a total area of 21,086 ha including 7713 ha of public forest. Small patches of private forest are also found in the landscape but they were not considered here as no field data were collected there. The public forest is largely dominated by pure stands of Scots pine ( *Pinus sylvestris*). Pure and mixed stands of oak ( *Quercus robur*) and beech are also found, but in a much smaller proportion.

The Snežnik forest is located in the Dinaric Mountains in southern Slovenia between 572 and 1792 m a.s.l. The Dinaric Mountains are a karst mountain range composed mainly of limestone and dolomite and characterised by an irregular and diverse topography and rockiness. The area has abundant precipitation (over 2000 mm annually on average), which is evenly distributed throughout the year. The average annual temperature is 6.5°C, with a mean monthly maximum temperature of around 16°C in July and a mean minimum of -3.4°C in January. The study area spans over 4725 ha and is almost completely covered by public forest (4660 ha). The main tree species are fir and beech, which are mostly found in uneven-aged mixed stands. Interestingly, in this study area, the upper forest limit is formed by beech stands and not conifer stands.

### General approach

**Figure 2.**
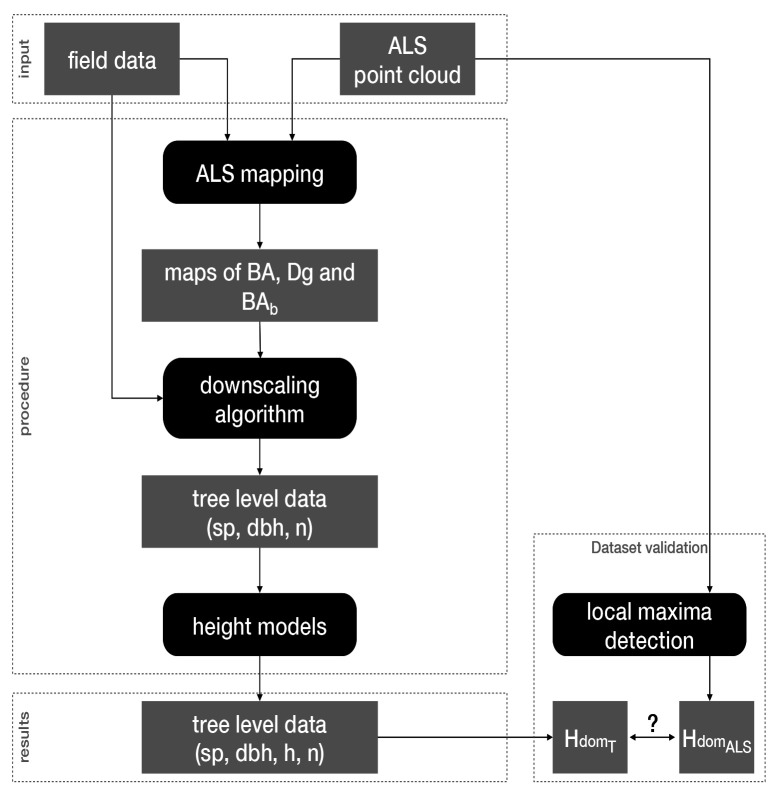
Workflow overview. Black boxes correspond to data generation steps feeding each other with datasets represented by grey boxes. BA: basal area; Dg: mean quadratic diameter; BA _b_: BA proportion of broadleaf trees; sp: species; dbh: diameter at breast height; h: height; n: number of trees; Hdom _ALS_ and Hdom _T_: stands dominant heights measured by ALS or calculated from the generated trees, respectively.

Here we outline the approach we adopted to produce the virtual landscapes corresponding to our three study areas ( Figure 2).

First, we produced raster maps of stand total basal area (BA), mean quadratic diameter (Dg) and proportion of broadleaf trees BA (BA _b_) at a 25 m resolution (see *ALS mapping*). For that, we used ALS point clouds along with field data (tree diameter and species identity). Thereafter, we generated trees in each 25x25 m ^2^ cell, specifying their diameter at breast height (dbh), number (n) and species (sp; see *Downscaling algorithm*). For that, we first assigned to each cell a stand from the field data based on the similarity of their BA, Dg and BA _b_ values (calculated as the Euclidean distance between each cell and each field plot in the three-dimensional space made up by the scaled values of BA, Dg and BA _b_). We then transformed the structure of the stand chosen from the field data (by changing the trees dbh, basal area and weight) to reach the BA and BA _b_ values of the cell. Finally, we used diameter-height models to assign heights (h) to all trees (see *Heights models*).

We evaluated the overall reliability of our workflow, i.e. its ability to produce virtual landscapes as close as possible to the real ones (see *Dataset validation*). In particular we carried out a leave-one-out cross validation (LOOCV) on our entire workflow. This analysis consisted in:

• comparing the observed and predicted values of BA, Dg, Ba _b_ and the quantiles of tree height and diameter;

• comparing the observed and predicted values of species abundance at the landscape level;

• calculating the frequency at which the most abundant species was correctly predicted at the cell level;

As a complement, we also compared the stands dominant heights measured by ALS (Hdom _ALS_) to those calculated from the trees we generated (Hdom _T_). Finally, we compared the spatial distribution of species to current expert knowledge.

### ALS mapping

The so-called "area-based” approach is a workflow commonly implemented for mapping stands variables in operational conditions [ White *et al.*, 2013]. It is based on the synergistic use of field plots and ALS point clouds. Estimation models for target forest variables are fitted with point clouds statistics, also called metrics, as predictor variables. Field plots are used for training the models. For the mapping step the predictor variables are computed in each cell of a raster layout over the whole acquisition area, and then the models are applied to obtain wall-to wall-estimates. This workflow was implemented in each study area.

***Forest areas.*** Reference areas for forest mapping were defined as the intersection of two layers for each site, one defining the administrative boundary and one defining the forest mask. Those extents are respectively:

- Bauges: the Geopark administrative extent with the forest mask defined by the BD Forêt v2 from the National Institute of Geographic and Forest Information [ IGN, 2019], excluding the “herbaceous”, “moors” and “ *Populus* plantations” categories;
- Milicz: the public forests of Milicz with the forest mask defined by the Forest Data Bank [ Bureau for Forest Management and Geodesy, 2020];
- Snežnik: the forest management units of Leskova Dolina and Snežnik with the forest mask defined by Snežnik-forest cover [ Service, 2020].

***Field data.*** In the Bauges, a local forest inventory with 320 plots was implemented in 2018. On each plot, all living trees with a dbh larger than 17.5 cm and within a 15 m horizontal distance from the plot centre had their dbh, position and species recorded. Trees with a dbh between 7.5 and 17.5 cm were counted according to simplified categories of diameter and species (coniferous / broadleaf). Plot centres were geolocated with survey-grade GNSS (Global Navigation Satellite System) receivers. Plots co-registration with the ALS data was improved when possible by comparing the positions of trees with the Canopy Height Model (CHM) derived from the point cloud.

At Milicz, a local forest inventory with 901 plots of 12.62 m radius was carried out in 2015. Species and diameter of all living trees with dbh above 7 cm were recorded. Plot centres were geolocated with survey-grade GNSS receivers.

At Snežnik, a total of 515 plots were inventoried, in 2013 for plots located in the Leskova Dolina management unit and in 2014 for plots located in the Snežnik management unit. Trees with a dbh above 30 cm within a 12.61 m distance from the plot centre had their diameter and species recorded. Trees with a dbh between 10 and 30 cm were recorded within a 7.98 m distance from the plot centre. Plot centres were geolocated with commercial-grade GNSS receivers.

The following stand-level variables were computed for each plot: total basal area (BA) in m ^2^.ha ^-1^, mean quadratic diameter (Dg) in cm and the proportion of broadleaf species in basal area (BA _b_). Weights were applied to correct for sampling intensity in the case of nested plots (Bauges and Snežnik).

***ALS data.*** The Bauges was covered by two ALS acquisitions with different settings and equipment. The southern part was covered between June and September 2016, the northern part in September 2018. Point densities computed at 25 m resolution in forest areas were respectively 5.9 ± 3.1 and 27.6 ± 13.3 m ^-2^. Intensity values were normalised by dataset, by subtracting the mean and dividing by the standard deviation of intensity values of points located inside the extent of field plots covered by each acquisition.

Milicz was covered by an ALS acquisition in August 2015. The point density was 16.5 ± 7.1 m ^-2^. The point cloud contains colour values extracted from aerial pictures with near infra-red, red and green bands.

Snežnik was covered by an ALS acquisition between February 14th and November 21st 2014. Forests might have been both in leaf-on and leaf-off conditions. The point density was 18.4 ± 10.1 m ^-2^. An ice storm occurred in Leskova Dolina management unit between January 30th and February 10th 2014. This event damaged the forest stands, and happened between the field inventory and the ALS acquisition. It affected the quality of the derived maps (see *Mapping*) and the realism of our virtual landscape (see *Dataset validation*).

***ALS metrics.*** All computations were performed with R software. Terrain metrics (aspect, elevation and slope) were computed by fitting a plane surface to points classified as ground. Before the computation of vegetation metrics, ALS point clouds were normalised, *i.e.* height above ground was computed for each point. Two types of metrics were then computed from the points classified as vegetation with a height above 2 meters (this limit was set to remove points of shrubs and low vegetation from the analysis):

- Point cloud metrics were directly computed from the point cloud using the aba_metrics function from the lidaRtRee R package. Those metrics summarise the geometry of the point cloud in a given area.
- Tree metrics were computed with the std_tree_metrics function from the characteristics of local maxima extracted from the CHM with the tree_segmentation function. CHM resolution was set to 0.5 m at Milicz, and 1 m at Snežnik and the Bauges due to higher variability of point density. Local maxima with a height lower than 5 m were discarded. Those metrics summarise the characteristics of trees detected in a given area of the point cloud. One of the tree metrics is the ALS dominant height (Hdom _ALS_), which is the mean height of the six highest local maxima. In case less than six maxima were present, the mean height of all maxima was used.

The metrics were computed for each field plot based on the point cloud located inside their extent, in order to build the dataset for model calibration (training step). The metrics were also computed in each 25×25 m ^2^ cell of the raster layout covering each acquisition, in order to build the prediction dataset (mapping step). Each metric map was visually checked for spatial patterns potentially linked to acquisition patterns, which eventually led to:

- discard some intensity-related metrics in Sneznik study area;
- remove ALS points acquired with a scan angle larger than 21 degrees in Milicz study area, in order to achieve a trade-off between metrics robustness, point density and comprehensive coverage of the study area.

***Models.*** For BA and Dg, we searched for the linear regression model that yielded the highest adjusted-R ^2^ with at most *n* = 6 independent variables among the above-mentioned ALS metrics. The model was given by:


$$\;\hat{y}=a_{0}+\sum_{i=1}^{n}a_{i}x_{i}\;(1)$$

with $\hat{y}$ the estimated value, ( *a _i_*) *i*∈{0,..., *n*} the model parameters and ( *x _i_*) *i*∈{1,..., *n*} the selected metrics. Two data transformations were also tested: a logarithm transformation of all variables and a Box-Cox transformation of the dependent variable. The logarithm transformation of all variables turns the model at Equation 1 into:


$$\;\hat{y}=e^{\left(a_{0}\right)}\times \prod_{i=1}^{n}x_{i}^{a_{i}}\;(2)$$

A bias correction factor had to be applied to the fitted values to obtain the predictions ( *P*):


$$\;P=\hat{y}\times e^{\left(\frac{v}{2}\right)}\;(3)$$

with *υ* the variance of the model residuals.

The Box-Cox transformation consists in determining the *λ* parameter that best normalises the distribution of the dependent variable ( *Y*). It is determined using the maximum likelihood-like approach of Box & Cox [1964] ( *powerTransform* function of car R package). *Y* is given by:


$$\;Y=\frac{\left(y^{\lambda }-1\right)}{\lambda }\;(4)$$

Equation 1 is then fitted with *Y* instead of *y*. The predictions *P* are obtained by applying the inverse Box-Cox transformation to the fitted values $\hat{Y}$ and a bias correction factor:


$$\;P={\left(\lambda \hat{Y}+1\right)}^{\frac{1}{\lambda }}\times \left(1+\frac{v}{2}\times \frac{1-\lambda }{(\lambda \hat{Y}+1{)}^{2}}\right)\;(5)$$

For broadleaf proportion (BA _b_), values are bounded to [0, 1]. A binomial generalised linear model with logit link was therefore fitted with the *glm R* function. The model was given by:


$$\;log\left(\frac{\hat{BA_{b}}}{1-\hat{BA_{b}}}\right)=a_{0}+\sum a_{i}x_{i}\;(6)$$

All metrics were at first included in the model and then a stepwise selection was used to reduce their number ( *stepAIC* function of the *MASS R* package).

***Stratification.*** When calibrating a statistical relationship between forest stand variables, which are usually derived from diameter measurements and ALS metrics, one relies on the hypothesis that the interaction of laser pulses with the leaves and branches structure is constant on the whole area. However, differences can be expected either due to variations in acquisition settings (flight parameters, scanner model), in forests (stand structure and composition) or in topography (slope). Better models might be obtained when calibrating stratum-specific relationships, provided each stratum is more homogeneous regarding the laser interaction with the vegetation. A trade-off has to be achieved between the within-strata homogeneity and the number of available plots for calibration in each stratum.

Depending on the study areas, different ancillary data are available for stratification. At the Bauges, two layers were used: species composition (mixed, broadleaf, coniferous) derived from the BD Forêt v2 and ALS survey. At Milicz, the following information was available for a total of 2175 stands: dominant species (coniferous, *Quercus*, other broadleaf) and stand age. At Snežnik, the following information was available for a total of 1536 stands: forest management unit (FMU: Snežnik or Leskova Dolina) and broadleaf proportion in volume, which is converted into a two (broadleaf or coniferous) or three-levels factor (adding the mixed category). The metrics selected in the 32 models for BA and Dg (which include at most six independent variables) are presented in Table S1 of the *Extended data*.

Field plots and raster cells were assigned to the category of the polygon which contains their centres.

***Mapping.*** Stratifications were compared based on expert knowledge taking into account the following criteria: minimum number of observations in strata, prediction error and number of variables in the model. The retained stratifications for the prediction models and the root mean square error (RMSE) of prediction estimated in leave-one-out cross validation are presented in Table 1.

**Table 1.** Stratification and root mean square error (RMSE) of predictions for the three study areas and three forest variables. BA: basal area (m ^2^.ha ^-1^); Dg: mean quadratic diameter (cm); BA _b_: broadleaf BA proportion (%).

study area	Variable	RMSE	Stratification: number and combinations
Bauges	BA	8.3	6: composition x ALS survey
	Dg	4.2	6: composition x ALS survey
	BA _b_	20.3	3: composition
Milicz	BA	5.4	7: (coniferous x 5 age classes), *Quercus sp*., other broadleaf
	Dg	3.7	3: coniferous, *Quercus sp*., other broadleaf
	BA _b_	12.9	2: coniferous, broadleaf
Snežnik	BA	9.6	4: FMU x composition (2 classes)
	Dg	7.6	6: FMU x composition (3 classes)
	BA _b_	19.3	2: FMU

**Figure 3.**
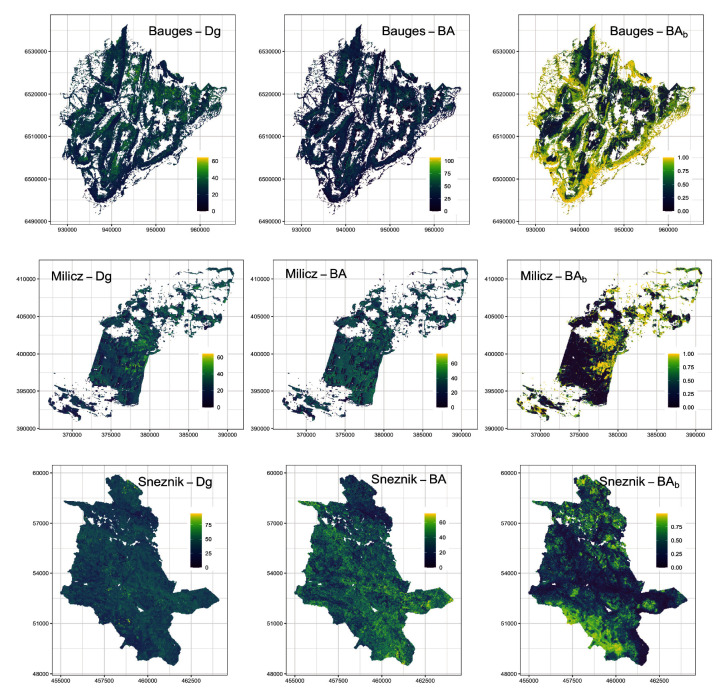
Airborne laser scanning (ALS) maps of forest variables for our three study areas at a 25 m resolution. Dg: mean quadratic diameter (cm), BA: basal area (m ^2^.ha ^-1^) and (BA _b_): proportion of broadleaf BA.

Prediction accuracy is better for mean diameter and lower for BA, which is common when estimated with ALS. Precision is quite low for broadleaf proportion, which could be expected as spectral data are usually better than ALS at classifying species. Prediction accuracy was higher at Milicz, intermediate at the Bauges and lower at Snežnik. Milicz was well suited for making predictions with its dense ALS data, homogeneous stands and precise co-registration. The Bauges has precise co-registration, but heterogeneous forest stands and two different ALS datasets. At Snežnik the data were much noisier, especially because of the ice storm event. The maps we created are presented in Figure 3.

### Downscaling algorithm

***Field data.*** At Milicz and Snežnik, we used the same dbh measurements as those used to calibrate the ALS models (from 901 plots at Milicz and from 515 plots at Snežnik, see *ALS mapping - Field data*). At the Bauges, we could not use the dbh measurements used to calibrate the ALS models because trees with a dbh smaller than 17.5 cm were not measured but counted by diameter classes. Instead, we used the tree diameter measurements from the 258 forest plots of the French National Forest Inventory (NFI) located in the study area. Those plots were inventoried between 2005 and 2018. They consist of three concentric plots of 6 m, 9 m and 15 m radius, where small (7.5 *<* dbh *<* 22.5 cm), medium (dbh *<* 37.5 cm) and big trees (dbh *>* 37.5 cm) were measured, respectively. At the Bauges, we used an additional information on forest vegetation: the map of forest types [ IGN, 2019], which we also used to delineate the forest areas (see *Forest areas*).

***Algorithm.*** Our algorithm consisted in associating to each 25×25 m ^2^ cell a field plot based on the similarity of their dendrometrical variables, and then in modifying the trees dbh, basal area and weight of this field plot in order to reach the total BA and the proportion of broadleaf BA (BAb) of the cell ( *i.e.* the values provided by the ALS maps). The algorithm breaks down as follows:

1. First, we calculated the total basal area (BA), mean quadratic diameter (Dg) and proportion of broadleaf BA (BA _b_) of all field plots.
2. Second, we associated to each 25×25 m ^2^ cell a field plot based on the similarity of their BA, Dg and BA _b_. These 3 variables were chosen for matching because together they provide a synthetic yet fairly accurate picture of the stands.
  (a) For this, we scaled the values of BA, Dg and BA _b_ between 0 and 1. We scaled the ALS and field data together to account for the possible differences in their range.
  (b) We then calculated the Euclidean distance between each cell and each field plot in the three-dimensional space made up by the scaled values of BA, Dg and BA _b_.
  (c) Finally, we associated to each cell the closest field plot in this three-dimensional space. For the Bauges study area, we assigned to each 25×25 m ^2^ cell a forest type ( *e.g.* pure beech, mixed deciduous forest, among others) from the map of forest types. We then associated the closest field plot sharing the same forest type to each cell.
3. Third, we transformed the field plots stand structure so that it matched the BA and BA _b_ values of the cells they were associated with.
  (a) For this, we first calculated *α*, a multiplier correction coefficient to be applied to all tree diameters of a field plot. The idea is to increase or decrease tree diameters so that their Dg reaches the Dg value of the cell to which they are associated. *α* is given by:
$$\;\alpha =\frac{Dg_{ALS}}{Dg_{F}}\;(7)$$
    with *Dg _ALS_* the Dg value of the cell given by the ALS mapping, and *Dg _F_* the Dg value calculated with the dbh of the trees from the field plot.
  (b) Thereafter, we calculated the weight (ω in n.ha ^-1^) of these trees with corrected diameters, so that the generated stand matches the BA and BAb values of the cell it is associated with. ω is given by:
$$\;\omega =\frac{40000^{1}}{\pi }\times \frac{ba_{tree_{ALS,F}}}{({\alpha \text{.}dbh}_{F}{)}^{2}}\;(8)$$
    where *dbh _F_* is the tree dbh in the field plot, and *ba _treeALS,F_* is the tree individual basal area derived from the ALS mapping and the field plot data using the following equation:
$$\;ba_{tree_{ALS,F}}=BA_{ALS}\times Prop_{{BC}_{\mathrm{ALS}}}\times Prop_{{Sp}_{F}}\times Prop_{tree_{F}}\;(9)$$
    where *BA _ALS_* is the total BA of the cell given by the ALS mapping, *Prop _BC _ALS__* is the BA proportion of broadleaf (resp. coniferous) trees given by the ALS mapping, *Prop _Sp _F__* is the BA proportion of species *Sp* in broadleaf (reps. coniferous) species in the field plot, and *Prop _tree _F__* is the BA proportion of this tree in species *Sp* in the field plot.
  (c) Finally, we divided *ω* by 16 to get the weight of the trees in the 25×25 m2 cells (ω being a weight per ha and 16 being the surface area ratio between 1 ha and a 25×25 m ^2^ cell). In doing so, the obtained tree weights can be either integer or decimal. However, the objective of our algorithm is to generate for each cell a list of individual trees with their associated diameter, height and species. From this perspective, decimal weights are not useful. We cannot simply round the tree weights to the nearest integer as this can lead to a significant over- or underestimation of the total number of trees in the cells. This is because the decimal part of the tree weights in the 25×25 m ^2^ cells is not the result of a random draw but directly depends on the surface area ratio between the field plot and the cell. As an example: 1 tree inventoried on a 400 m ^2^ field plot will always obtain a weight of 1.56 in a 25 × 25 m ^2^ cell, and a weight of 2 after rounding to the nearest integer. In order to obtain integer tree weights in the 25 × 25 m ^2^ cells while avoiding this bias, we performed a Bernoulli draw on the decimal part of the tree weights. As an example, a weight of 1.56 has a 56% chance of becoming 2, and a 44% chance of becoming 1. As this rounding of the weights slightly modifies the total BA of the generated stand, we transformed again the trees dbh to reach the total BA provided by the ALS mapping using the trees BA and their integer weights ( *ω _int_*) as follows:
$$\;dbh_{final}=\sqrt{\frac{40000^{1}}{\pi }\times \frac{\frac{ba_{tree_{ALS,F}}}{16}}{\omega_{int}}}\;(10)$$
    As this last transformation only compensates for the rounding, the changes in dbh are minor.

This procedure has multiple benefits (see proofs in *Extended data*): it makes it possible to reach the BA and BA _b_ values given by the ALS mapping. It also maintains the Dg ratios observed on the field plots between the different species. The Bernouilli draw used to get integer tree weights only adds a minor variability. We created the three virtual landscapes by applying this algorithm to each study area separately.

### Heights models

We developed individual diameter-height models for the three study areas to assign heights to all generated trees.

***Field data.*** At Snežnik and Milicz, the diameter and height measurements come from the same field plots used for the ALS models calibration (see *ALS mapping - Field data*). At the Bauges, no height measurements were collected in the field plots used to calibrate the ALS models. We therefore used the tree diameter and height measurements of the 240 French NFI plots located in the study area (inventoried between 2005 and 2016). At Milicz and the Bauges, the heights were measured for all species in all diameter classes. At Snežnik, tree heights were measured only on two to four trees from the upper layer. The number of trees with both diameter and height measurements in each study area is summarised per species in Table 2.

**Table 2.** Number of trees for the diameter-height models calibration in each study area and for each species. For each study area, all the species with less than 100 observations are grouped into the "other species” category.

Species	Number of trees for			
	Bauges	Milicz	Snežnik
*Abies alba*	468		638
*Acer pseudoplatanus*	181	228	
*Alnus glutinosa*		823	
*Betula pendula*		1 519	
*Carpinus betulus*		808	
*Fagus sylvatica*	705	2 199	435
*Fraxinus excelsior*	209		
*Larix decidua*		709	
*Picea abies*	551	2 183	325
*Pinus sylvestris*		24 995	
*Prunus serotina*		191	
*Quercus petraea*	130		
*Quercus rubra*		308	
*Quercus* undefined *		1 916	
*Tilia cordata*		311	
Other species	642	522	29
TOTAL	2 886	36 712	1 427

*At Milicz, the *Quercus* undefined is mainly *Quercus robur*.

***Models.*** We used a mixed effect model to predict individual tree height from the ratio between the tree dbh and the stand Dg (to account for the tree social status) and from the stand Dg (to account for the stand development stage). We considered the site effect as a random effect. Finally, as the variance of height increases with height due both to increasing measurement errors and to individual cumulative variations, we accounted for heteroscedasticity by modelling the error term with a power of the fitted values. The model is given by:


$$h_{tot}=1.3+(1+\alpha_{site})\times \alpha_{sp}\times \left(1-e^{\left(-\alpha_{1}\times Dg^{\alpha_{2}}\right)}\right)\times {\left(1-e^{\left(-\beta_{sp}\times \frac{dbh}{Dg}\right)}\right)}^{\gamma }+\;(11)$$

where *α _sp_*, *α*_1_, *α*_2_, *β _sp_* and *γ* are parameters to be estimated; and *α _site_*, a random effect accounting for the site effect. This model has an asymptotic form: *α _sp_* corresponds to the species-specific asymptotic value, and *β _sp_* is the species-specific speed for reaching the asymptotic value.

At Snežnik, most of the trees selected for height measurement were dominant or co-dominant trees. Moreover, more than half of the plots only had two observations. This precludes to fit the part of the curve with small diameters within the stand. We solved this issue by assuming that the within-stand relationship at the Bauges was similar at Snežnik, as these landscapes are quite similar in terms of species, stand structure (mostly uneven-aged), or elevation (mountains). Therefore, for Snežnik height predictions, we used the *β _sp_* and *γ* fitted values of the Bauges model.

We fitted one mixed effect model for each study area using the *nlme* function from the *nlme R* package. We modelled the residual errors using a *varPower* function of the fitted values. The parameters are presented in Table 3, Table 4, and Table 5 for the three study areas.

**Table 3.** Parameters of the Bauges diameter-height model.

Parameter	Value	Standard error	p-value
*α _Fa.sy._*	41.05595	4.3	*<*10 ^–3^
*α _Pi.ab._*	55.11821	5.8	*<*10 ^–3^
*α _Ab.al._*	48.46640	5.1	*<*10 ^–3^
*α _Fr.ex._*	40.94293	4.3	*<*10 ^–3^
*α _Ac.ps._*	37.95001	4.0	*<*10 ^–3^
*α _Qu.pe._*	36.64676	4.2	*<*10 ^–3^
*α _OtherSp._*	36.87834	3.8	*<*10 ^–3^
*α*_1_	0.01594	0.0030	*<*10 ^–3^
*α*_2_	1.26326	0.10	*<*10 ^–3^
*β _Fa.sy._*	1.71474	0.08	*<*10 ^–3^
*β _Pi.ab._*	0.99226	0.05	*<*10 ^–3^
*β _Ab.al._*	1.17894	0.06	*<*10 ^–3^
*β _Fr.ex._*	2.01951	0.12	*<*10 ^–3^
*β _Ac.ps._*	2.08068	0.12	*<*10 ^–3^
*β _Qu.pe._*	1.56216	0.16	*<*10 ^–3^
*β _OtherSp._*	1.84067	0.08	*<*10 ^–3^
*γ*	1.42595	0.05	*<*10 ^–3^
Power of the variance model			0.51
Standard deviation of the plot level random effect			0.14
Standard deviation of residual error			0.59

**Table 4.** Parameters of the Milicz diameter-height model.

Parameter	Value	Standard error	p-value
*α _Pi.sy._*	48.55802	2.3	*<*10 ^–3^
*α _Fa.sy._*	48.01692	2.3	*<*10 ^–3^
*α _Pi.ab._*	60.35196	3.1	*<*10 ^–3^
*α _Qu.un._*	52.24210	2.5	*<*10 ^–3^
*α _Be.pe._*	51.60844	2.5	*<*10 ^–3^
*α _Al.gl._*	49.34039	2.4	*<*10 ^–3^
*α _Ca.be._*	36.73985	1.8	*<*10 ^–3^
*α _La.de._*	52.06992	2.5	*<*10 ^–3^
*α _Ti.co._*	45.25535	2.4	*<*10 ^–3^
*α _Qu.ru._*	45.74754	2.4	*<*10 ^–3^
*α _Ac.ps._*	41.50894	2.2	*<*10 ^–3^
*α _Pr.se._*	36.18532	2.9	*<*10 ^–3^
*α _OtherSp._*	54.94652	2.8	*<*10 ^–3^
*α*_1_	0.01958	0.001	*<*10 ^–3^
*α*_2_	1.13831	0.035	*<*10 ^–3^
*β _Pi.sy._*	2.73192	0.024	*<*10 ^–3^
*β _Fa.sy._*	1.98085	0.032	*<*10 ^–3^
*β _Pi.ab._*	1.20700	0.035	*<*10 ^–3^
*β _Qu.un._*	1.62943	0.027	*<*10 ^–3^
*β _Be.pe._*	2.11097	0.037	*<*10 ^–3^
*β _Al.gl._*	2.04760	0.045	*<*10 ^–3^
*β _Ca.be._*	2.86677	0.063	*<*10 ^–3^
*β _La.de._*	2.33369	0.050	*<*10 ^–3^
*β _Ti.co._*	1.89682	0.064	*<*10 ^–3^
*β _Qu.ru._*	2.38748	0.095	*<*10 ^–3^
*β _Ac.ps._*	2.56340	0.102	*<*10 ^–3^
*β _Pr.se._*	2.04373	0.150	*<*10 ^–3^
*β _OtherSp._*	1.50792	0.019	*<*10 ^–3^
*γ*	1.55264	0.040	*<*10 ^–3^
Power of the variance model			0.16
Standard deviation of the plot level random effect			0.09
Standard deviation of residual error			1.09

**Table 5.** Parameters of the Snežnik diameter-height model.

Parameter	Value	Standard error	p-value
*α _Ab.al._*	66.17413	5.4	*<*10 ^–3^
*α _Fa.sy._*	53.81402	4.4	*<*10 ^–3^
*α _Pi.ab._*	76.82544	6.3	*<*10 ^–3^
*α*_1_	0.0251	0.0036	*<*10 ^–3^
*α*_2_	1.00672	0.075	*<*10 ^–3^
*β _Ab.al._* *	1.17894	* taken from the Bauges model	
*β _Fa.sy._* *	1.71474		
*β _Pi.ab._* *	0.99226		
γ*	1.42595		
Power of the variance model			-0.56
Standard deviation of the plot level random effect			0.077
Standard deviation of residual error			15.8

## Dataset validation

### Method

We carried out a leave-one-out cross validation (LOOCV) to evaluate the realism of the virtual landscapes we generated. This consisted in excluding a field plot from our entire workflow and comparing the predicted values obtained to the observed values. This operation was repeated within each landscape for all field plots. We calculated the root mean square error (RMSE) of the predictions of BA, Dg, Ba _b_ and the quantiles of tree height and diameter. As part of the LOOCV, we also compared the observed and predicted values of species abundance at the landscape level (in BA) and calculated the frequency at which the most abundant species was correctly predicted at the cell level.

As a general validation of our approach, we compared the stand dominant heights estimated by ALS (Hdom _ALS_) to those calculated from the trees we generated (Hdom _T_). We expect Hdom _ALS_ to be as close to reality as possible, as tree height is among the most reliable ALS measurement [ Van Leeuwen & Nieuwenhuis, 2010] and can be derived from ALS data with little processing and no field data. Hdom _ALS_ therefore serves here as a reference to which Hdom _T_ is compared.

In practice, Hdom _T_ is calculated as the mean height of the six highest trees, while Hdom _ALS_ is calculated as the mean height of the six highest local maxima (see *ALS metrics*). In case less than six trees/maxima were found, the mean height of all trees/maxima was used. These dominant heights are calculated at the 25×25 m ^2^ cell level. There is some circularity in comparing HdomALS and HdomT as models predicting BA, Dg and BA _b_ from ALS point clouds may include ALS derived height metrics or more generally metrics which are correlated with the dominant height estimated from ALS point clouds. The results of this comparison must therefore be interpreted with caution.

Finally, we examined the spatial distribution of species at each site and compared it to current expert knowledge.

### Results

Overall, the virtual landscapes are in good agreement with the landscapes they aim to reproduce. The generated stand structures and compositions are consistent with the observations and make it possible to distinguish stands at different stages of development and with different compositions.

**Figure F1.**
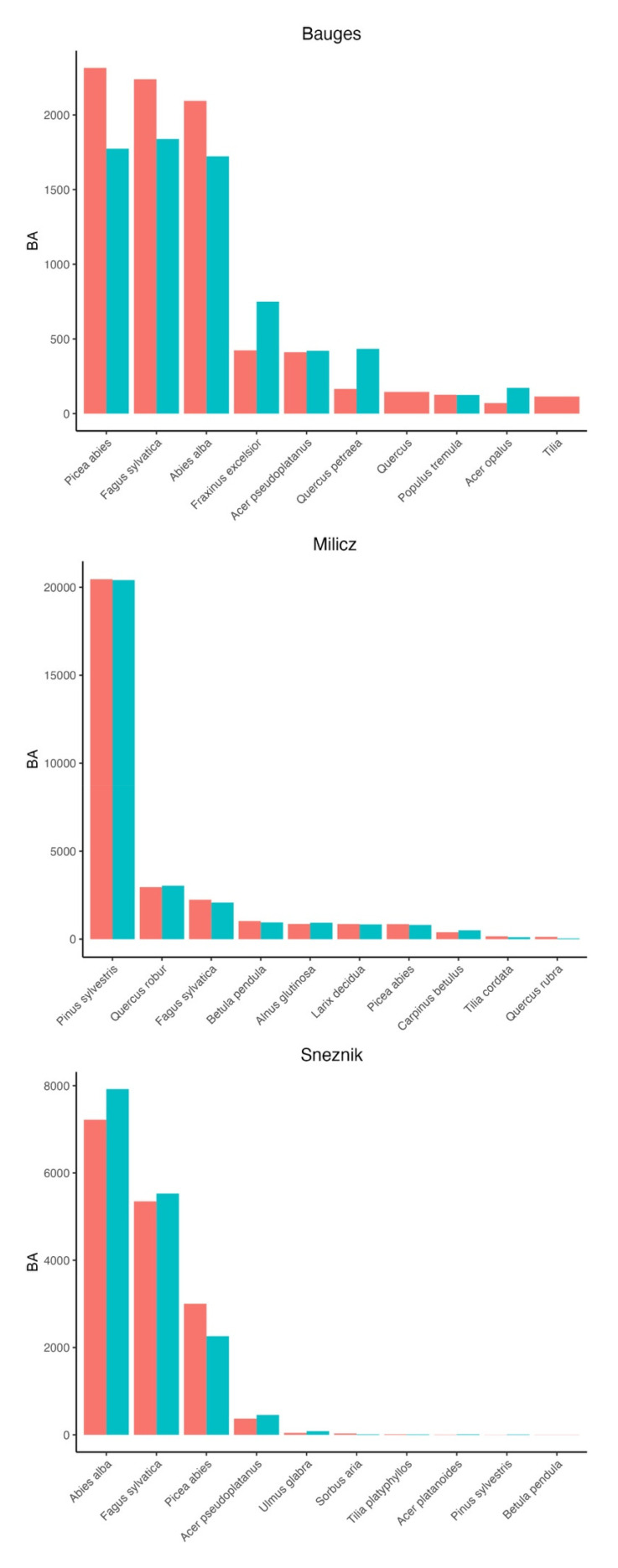
Predicted (blue) and observed (red) species abundance in BA (m ^2^) at the landscape level. In the Bauges, we only considered trees with a dbh greater than 17.5, as smaller trees were not identified in the local forest inventory (LFI) but only grouped in two categories (coniferous and broadleaf). Also, some predictions are missing in the Bauges because some trees in the LFI were not identified at the species level and therefore can’t find a match in the generated trees which all receive a species name.

At Milicz, predictions are the most accurate. The RMSE of all evaluated variables are the lowest in comparison with the other landscapes ( Table T1). Species abundance at the landscape level is also better reproduced (Figure F1). Finally, in 76.2% of cases, the generated main species corresponds to the observed main species. This higher quality of predictions can be explained by the fact that Milicz has the highest density of inventory plots and the least complex landscape, with a predominance of even-aged monospecific stands and the lowest species diversity among our three landscapes.

**Table T1.** Root mean square error (RMSE) of predictions for the three study areas obtained from the leave-one-out cross validation (LOOCV) carried out on our entire workflow. BA: basal area (m ^2^.ha ^-1^); Dg: mean quadratic diameter (cm); BA _b_: broadleaf BA proportion (%); dbh: diameter at breast height (cm); h: tree height (m); Q _0.5_ and Q _0.95_: fiftieth and ninety-fifth percentiles, respectively, of the distribution of dbh and h. dbhQ _0.5_ is not considered as it is almost similar to Dg. The RMSE values from the LOOCV of ALS models presented in Table 1, are added here in brackets to facilitate comparisons. At Sneznik, RMSE of hQ _0.5_ could not be calculated as only dominant trees were measured on the field. At the Bauges, RMSE of hQ _0.5_ and hQ _0.95_ could not be calculated as no tree heights were measured in the field plots used to calibrate the ALS models.

Study area	Variable	RMSE
Bauges	BA	9.5 (8.3)
	Dg	5.4 (4.2)
	BA _b_	21.6 (20.3)
	dbhQ _0.95_	16.1
	hQ _0.5_	-
	hQ _0.95_	-
Milicz	BA	5.4 (5.4)
	Dg	3.9 (3.7)
	BA _b_	13.1 (12.9)
	dbhQ _0.95_	8.8
	hQ _0.5_	5.0
	hQ _0.95_	2.6
Sneznik	BA	9.6 (9.6)
	Dg	7.9 (7.6)
	BA _b_	20.0 (19.3)
	dbhQ _0.95_	13.1
	hQ _0.5_	-
	hQ _0.95_	4.7

At the Bauges and Sneznik, the RMSE of the evaluated variables are comparable ( Table T1.). In contrast, predictions of species abundance at the landscape level are more accurate at Sneznik ( Figure F1). The same applies to the compositions predicted at the plot level: the predicted main species corresponds to the observed main species in 63.1% of cases at Sneznik and in 37.2% of cases at the Bauges. However, two datasets were used in the Bauges. In the local forest inventory (LFI) not all trees were identified at the species level and trees with a dbh between 7.5 and 17.5 cm were not measured but counted by diameter classes and grouped in two categories (coniferous and broadleaf). This led us to use a local subset of the NFI from which composition is derived in our downscaling algorithm. The poorer composition predictions in the Bauges might therefore partly be an artefact arising from the evaluation itself, as the LFI may not be suitable to serve as a field reference.

The fact that the RMSE values obtained from the LOOCV carried out on our entire workflow are almost similar to the RMSE values obtained from the LOOCV of ALS models shows that the downscaling algorithm hardly adds any error ( Table 1, Table T1). The main way of increasing the realism of our virtual landscapes would therefore be to improve the ALS models.

**Figure 4.**
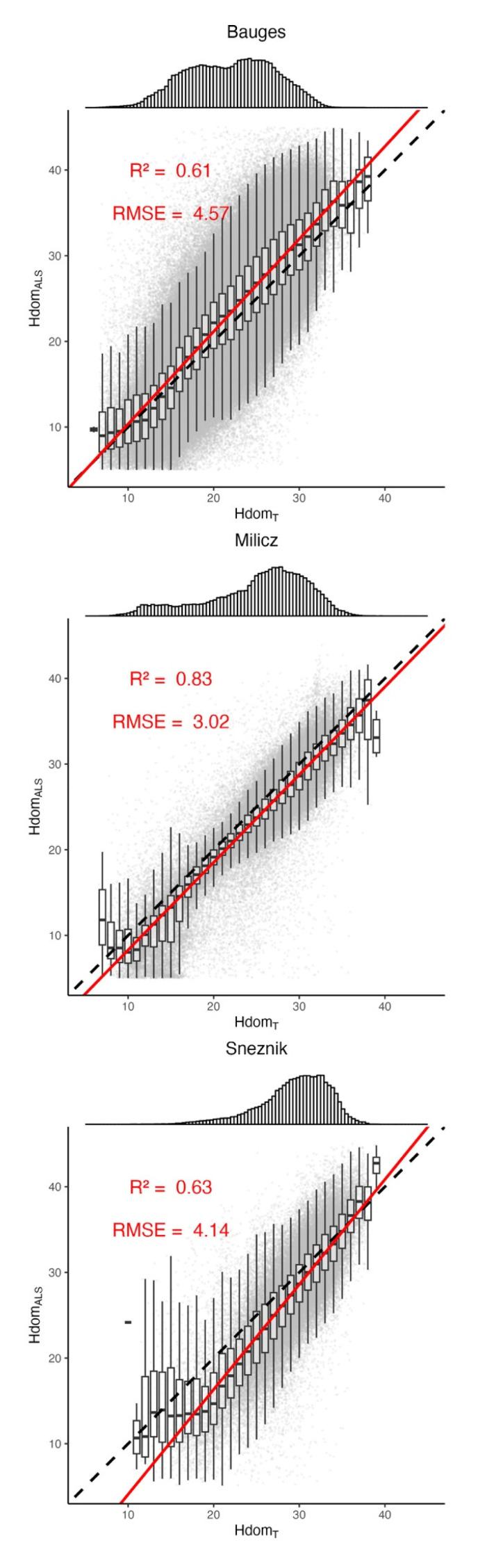
Comparison of the stands dominant heights measured by ALS (Hdom _ALS_, in m) to those calculated from the generated trees (Hdom _T_, in m). The top panels show the distribution of Hdom _T_. The dashed lines indicate the y = x line. The red lines correspond to the regression lines. The root mean square error (RMSE) values between Hdom _ALS_ and Hdom _T_, as well as the regression R-Squared values are shown in red.

With R ^2^ values ranging from 0.61 to 0.83 ( Figure 4) and RMSE values below 5 m, HdomALS and HdomT are consistent with one another. This provides a general validation of our workflow. As discussed above, the better predictions obtained at Milicz might steam from the higher density of inventory plots and the lower complexity of the landscape. At Sneznik, HdomT tends to be overestimated as HdomALS decreases. This divergence could be due to the ice storm that occurred between the field inventory and the ALS acquisition and that might have biased the ALS models.

Overall, species spatial distribution in the virtual landscapes is consistent with field observations. In the Bauges, pure and mixed stands of fir and spruce are more abundant at higher elevation while mixed stands of broadleaf species are found at lower elevation. At Milicz, pure stands of Scots pine are found at lower elevation while broadleaf species and mixed stands appear at higher elevation. Finally, at Sneznik, pure beech stands are found at higher elevation while fir is found at lower elevation in pure or mixed stands (a specific feature of the site).

Our procedure is not free of flaws and some outliers are present in the generated data (i.e. stands with extreme values of BA, Dg, tree height or density). These outliers are a direct consequence of the uncertainties associated with the models we used. The realism of the stands associated with these extreme values is open to question. However, separating realistic from unrealistic stands seems difficult as extreme values can be locally observed. It is therefore up to the users of the dataset to decide whether or not to consider these stands depending on their objectives.

## Virtual landscapes overview

**Figure 5.**
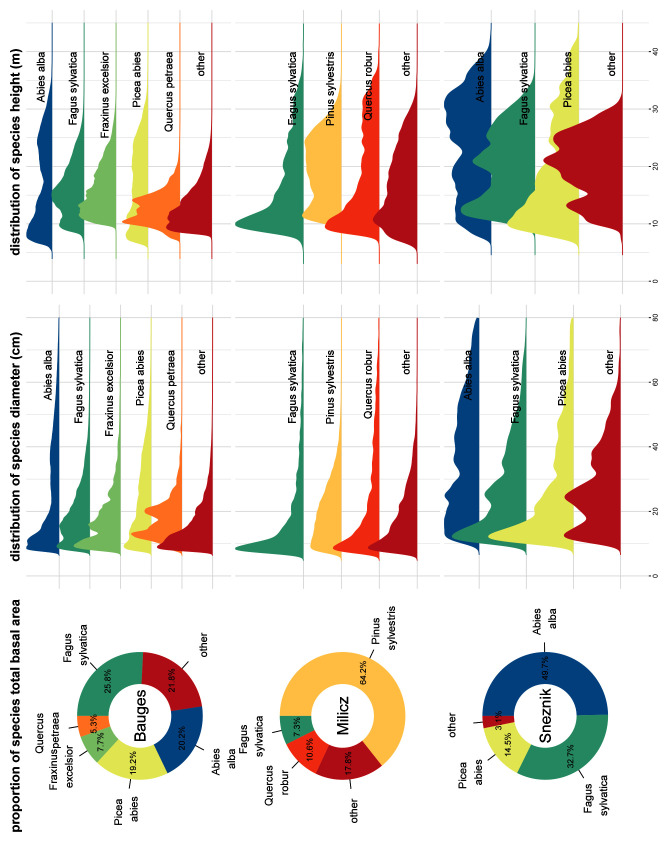
Main species basal area proportion, diameter distribution and height distribution in the three virtual landscapes. Species accounting for less than 5% of the virtual landscapes total basal area were grouped in the ’other’ category.

Overall, 42,394,479 trees belonging to 51 different species were generated: 35,134,985 trees of 40 different species were generated at the Bauges, 5,726,420 trees of 32 different species at Milicz and 1,533,074 trees of 16 different species at Snežnik. The main species BA proportion as well as their h and dbh distributions are shown in Figure 5 for each virtual landscape.

## Data Availability

**Bauges** The maps of forest types (BD Forêt®V2) are available to download from the National Institute for Geographic and Forestry Information website at https://geoservices.ign.fr/bdforet, under the Etalab open license 2.0. The French National Forest Inventory data are available to download from the National Institute for Geographic and Forestry Information website at https://inventaire-forestier.ign.fr/dataifn/, under the Etalab open license 2.0. The local forest inventory dataset is available for non-commercial use upon request to Jean-Matthieu Monnet ( jean-matthieu.monnet@inrae.fr). A data sharing agreement will have to be established, with the following restrictions: data are available for internal use only and cannot be distributed; results obtained from the data can be displayed or distributed provided they do not allow the estimation of growing stock in individual private properties; data funding (Ademe grant 1703C0069) should be cited. ALS data in the northern part (Haute-Savoie) are available to download from the Recherche Data Gouv dataverse at https://doi.org/10.57745/ZUT1MJ, under the Etalab open license 2. ALS data in the southern part (Savoie) can be purchased upon request to (Régie de Gestion des Données Savoie Mont Blanc) at https://www.rgd.fr/. **Milicz** The stand data in the ESRI Shapefile format are available to download from the Polish Forest Data Bank at https://www.bdl.lasy.gov.pl/portal/wniosek-en. The local forest inventory dataset and ALS data are available for non-commercial use upon request to Jarosław Socha ( jaroslaw.socha@urk.edu.pl). A data sharing agreement will have to be established, with the following restrictions: data are available for internal use only and cannot be distributed; data funding (REMBIOFOR - BIOSTRATEG1/267755/4/NCBR/2015) should be cited. **Sneznik** The forest inventory data (in *.xlsx and *.shp formats) and maps of forest types and species mixture (in *.shp format) are available upon request to Slovenia Forest Service ( zgs.tajnistvo@zgs.si; rok.pisek@zgs.si). A data sharing agreement will have to be established, with the following restrictions: data are only available for the study that is the subject of the agreement; Slovenia Forest Service should be acknowledged for providing the data in all publications. ALS data are available to download from the Slovenian Environment Agency website at http://gis.arso.gov.si/evode, under the terms of the international Creative Commons 4.0 license ( http://www.evode.gov.si/fileadmin/user_upload/Lidar_pogoji_uporabe.pdf): the data user must indicate the data source at each publication of data or products, specifying ”Slovenian Environmental Agency, type of data and period to which the data refer or the date of the database”. Zenodo: I-MAESTRO data: 42 million trees from three large European landscapes in France, Poland and Slovenia. https://doi.org/10.5281/zenodo.7462440 [ Aussenac *et al.*, 2022]. For each virtual landscape we provide a table (in .csv format) with the following columns: cellID25: the unique ID of each 25x25 m ^2^ cell sp: species latin names n: number of trees. n is an integer >= 1, meaning that a specific set of species “sp”, diameter “dbh” and height “h” can be present multiple times in a cell. dbh: tree diameter at breast height (cm) h: tree height (m) We also provide, for each virtual landscape, a raster (in .asc format) with the cell IDs (cellID25) which makes data spatialisation possible. The coordinate reference systems are EPSG: 2154 for the Bauges, EPSG: 2180 for Milicz, and EPSG: 3912 for Sneznik. We provide Table S1 presenting the metrics used in the 32 stratum-specific prediction models of BA and Dg. Finally, we provide a proof of how, in the downscaling algorithm, multiplying the trees dbh by the *α* correction coefficient makes it possible to reach the cells BA value derived from the ALS mapping.
