# Supplementary material for: Diameter, height and species of 42 million trees in three European landscapes generated from field data and airborne laser scanning data
Source: Open Res Eur. 2023 Dec 5;3:32. Originally published 2023 Feb 14. [Version 2] doi: 10.12688/openreseurope.15373.2 (PMC10823913; doi:10.12688/openreseurope.15373.2)
Supplement: Peer review history

# Reviewer report: Reviewer response for version 2

**DOI:** 10.21956/openreseurope.18268.r36612

**Published:** 2024-01-24

**Authors:** Fabian Fischer

**Affiliations:** 1 School of Biological Sciences, University of Bristol, Bristol, England, UK

The authors have done an impressive job addressing the reviewers' comments and the paper now presents an intriguing study of how one could produce large-scale inventory maps. The validation procedure, in particular, is now comprehensive and, by relying on leave-one-out cross validation (LOOCV) across the whole workflow, provides readers with a clear idea of the quality of the product.

 I have only a few minor comments:

- R packages should generally be cited in the references, but this seems not the case (I could not find references for, e.g, *MASS *package, *lidaRtRee *package)
- From my understanding, the whole workflow was applied separately at each site, but this is not immediately clear when reading the paper. It would be worth mentioning this early on somewhere in the methods section.
- For the validation tables (Tables 1 and T1), it would have been nice to also see a relative RMSE (rRMSE), i.e., RMSE divided by the standard deviation or mean of the corresponding variable. This would help readers understand whether these prediction errors are large or not, make BA/D _g_/BA _b_ errors comparable between each other and also make estimates better comparable across sites
- Generally speaking, I found that there was not much information on the ALS data processing. How was ground classification done? Did you remove noise? Is it all done with the *lidaRtRee* R package? This is not hugely important for your study, and if it was all done already by the data providers and with a variety of methods, you could also just state that.

Are sufficient details of methods and materials provided to allow replication by others?

Yes

Is the rationale for creating the dataset(s) clearly described?

Yes

Are the datasets clearly presented in a useable and accessible format?

Yes

Are the protocols appropriate and is the work technically sound?

Partly

Reviewer Expertise:

My areas of expertise are in lidar processing, individual-based modelling, as well as the creation of simulated forest stands (cf. my 2020 paper on this topic, mentioned in the review), which is very close to what the authors have been working on.

I confirm that I have read this submission and believe that I have an appropriate level of expertise to confirm that it is of an acceptable scientific standard.

# Reviewer report: Reviewer response for version 2

**DOI:** 10.21956/openreseurope.18268.r36613

**Published:** 2023-12-29

**Authors:** Nikolai Knapp

**Affiliations:** 1 Thünen Institute of Forest Ecosystems, Eberswalde, Germany

Thank you for revising the manuscript, for adding further validation and for the detailed answers to the comments. I only have one further remark: It would be good to mention the used lidar metrics explicitly, either by summarizing them in the text or listing them in a table. If they are only referenced as functions in the lidaRtRee package, readers have to search in the package documentation and, more importantly, the approach may become unreproducible, in case the package changes in the future and the used metrics were not documented. Otherwise, I congratulate the authors on this well written manuscript and I am looking forward to future applications of the method.

Are sufficient details of methods and materials provided to allow replication by others?

Yes

Is the rationale for creating the dataset(s) clearly described?

Yes

Are the datasets clearly presented in a useable and accessible format?

Yes

Are the protocols appropriate and is the work technically sound?

Yes

Reviewer Expertise:

Forest monitoring, forest modeling, lidar remote sensing

I confirm that I have read this submission and believe that I have an appropriate level of expertise to confirm that it is of an acceptable scientific standard.

# Reviewer report: Reviewer response for version 1

**DOI:** 10.21956/openreseurope.16618.r31138

**Published:** 2023-05-12

**Authors:** Nikolai Knapp

**Affiliations:** 1 Thünen Institute of Forest Ecosystems, Eberswalde, Germany

The paper presents an innovative approach to generate forest stand structure information at landscape extent and single tree resolution based on airborne lidar data and inventory plots. The approach is not based on individual tree detection (ITD) from lidar, but operates in an area-based (ABA) fashion at 25 m x 25 m cell scale. The inventory plots serve as lookup tables. Structure metrics are being estimated for every cell in the landscape based on lidar metrics. Then, each cell is being assigned to the most similar stand from the inventory lookup table based on a minimum distance of a set of structure metrics. The dbh values of the trees are then adjusted according to a proposed algorithm, such that the final structure metrics match the ones predicted for the cell. Finally, the generated forest landscapes are being validated by calculating dominant height for each cell based on the generated stands and comparing them to dominant heights directly obtained from lidar. The approach has been applied to three different regions in France, Poland and Slovenia.

 The presented approach is very interesting and useful as an efficient solution to generate maps at single tree resolution and landscape extent, which are highly relevant, e.g., for spatial and temporal interpolation of forest inventories and for modelling tasks. The method is well documented and the case studies along with the provided datasets make it an innovative publication. However, I have listed some comments below, which the authors should consider during revision.

**Detailed comments:**

- In the Abstract, I suggest to remove the tilde signs from 100~000~ha.
- On page 4 “For that, we first assigned to each cell a stand from the field data based on the similarity of their BA, Dg and BAb values.” it should already be briefly mentioned how “similarity” is defined, i.e. minimum distance of normalized values.
- I suggest to mention earlier (in the Abstract or Introduction), that the study follows an ABA approach, because readers might expect an ITD approach, if the final product are landscapes at tree level.
- Why were BA and Dg chosen as the structure metrics for matching? Would it not be important to also consider metrics that capture stem size heterogeneity / stem size distribution?
- On page 6, what is meant by “Point cloud metrics were directly computed from the point cloud or(?) from the derived CHM”? I suggest to list all lidar metrics which were used in a table.
- In Table 1, why are RMSE values for BAb > 1? In case they are given in percent, please add “(%)” to the caption.
- On page 9, the multiplication by 40000/pi and the division by 16 need to be explained. I suspect they convert values to the 1 ha and then back to the 25-m scale, however these scale factors should be explained explicitly. Also, the purpose of the rounding under “c)” should be better explained.
- Figure 4: What is the explanation for the seemingly better fit (higher R²) in Milicz compared to Bauges?

**General comments (for a possible Outlook):**

- Unlike an ITC approach, the presented method does not provide precise tree positions within the 25-m cells. Are there ways to expand the approach to additionally generate tree positions?
- Would it be possible/useful to add a height correction algorithm based on ALS heights (local maxima), similar to the dbh adjustment algorithm?

**Comments about the data:**

- The information about the coordinate reference system is missing. I was not able to georeference the asc files in a GIS.

It would be better to use unique file names, e.g. “milicz_cellID25.asc” etc. to be able to load all rasters in one GIS session.

Are sufficient details of methods and materials provided to allow replication by others?

Yes

Is the rationale for creating the dataset(s) clearly described?

Yes

Are the datasets clearly presented in a useable and accessible format?

Yes

Are the protocols appropriate and is the work technically sound?

Yes

Reviewer Expertise:

Forest monitoring, forest modeling, lidar remote sensing

I confirm that I have read this submission and believe that I have an appropriate level of expertise to confirm that it is of an acceptable scientific standard, however I have significant reservations, as outlined above.

# Response

**Published:** 2023-11-24

**Authors:** Raphaël Aussenac

**Affiliations:** CIRAD, France

**We would like to thank the reviewers for their useful comments which have helped significantly improve the manuscript. A special effort has been made to provide an in-depth evaluation of the generated landscapes, to clarify the functioning of our downscaling algorithm and to consolidate the rational of our work.**

**This revision has led us to optimise our code (better memory management, more effective parallelisation, etc.). It is now much faster, taking less than 1 hour to generate the 42 million trees of our three landscapes, while it used to take “less than 5 hours” to generate the 35 million trees of the Bauges landscape. We updated the introduction accordingly.**

**Please find below our replies to your comments.**

 The paper presents an innovative approach to generate forest stand structure information at landscape extent and single tree resolution based on airborne lidar data and inventory plots. The approach is not based on individual tree detection (ITD) from lidar, but operates in an area-based (ABA) fashion at 25 m x 25 m cell scale. The inventory plots serve as lookup tables. Structure metrics are being estimated for every cell in the landscape based on lidar metrics. Then, each cell is being assigned to the most similar stand from the inventory lookup table based on a minimum distance of a set of structure metrics. The dbh values of the trees are then adjusted according to a proposed algorithm, such that the final structure metrics match the ones predicted for the cell. Finally, the generated forest landscapes are being validated by calculating dominant height for each cell based on the generated stands and comparing them to dominant heights directly obtained from lidar. The approach has been applied to three different regions in France, Poland and Slovenia.

 The presented approach is very interesting and useful as an efficient solution to generate maps at single tree resolution and landscape extent, which are highly relevant, e.g., for spatial and temporal interpolation of forest inventories and for modelling tasks. The method is well documented and the case studies along with the provided datasets make it an innovative publication. However, I have listed some comments below, which the authors should consider during revision.

 Detailed comments:  

 In the Abstract, I suggest to remove the tilde signs from 100~000~ha. **Done as suggested**

 On page 4 “For that, we first assigned to each cell a stand from the field data based on the similarity of their BA, Dg and BAb values.” it should already be briefly mentioned how “similarity” is defined, i.e. minimum distance of normalized values. **We added a definition of similarity at the end of the sentence as follows: “For that, we first assigned to each cell a stand from the field data based on the similarity of their BA, Dg and BA _b_ values (calculated as the Euclidean distance between each cell and each field plot in the three-dimensional space made up by the scaled values of BA, Dg and BA _b_).”**  

 I suggest to mention earlier (in the Abstract or Introduction), that the study follows an ABA approach, because readers might expect an ITD approach, if the final product are landscapes at tree level. **Thank you for pointing that out. To make that point clear from the beginning, we now specify in the abstract that we use an ABA: “Tree diameter, height and species data were generated combining field data, vegetation maps and airborne laser scanning (ALS) data following an area-based approach.”** **To be consistent throughout the article, we also replaced, in the ALS mapping section, “area-based method” for “area-based approach.” ** 

 Why were BA and Dg chosen as the structure metrics for matching? Would it not be important to also consider metrics that capture stem size heterogeneity / stem size distribution? **BA and Dg were chosen because together they capture the development stage of stands. Along with BA _b_, they provide a synthetic yet fairly accurate picture of the stands and seem therefore appropriate for our matching procedure. We added this point in the Algorithm section as follows: “2. Second, we associated to each 25×25 m ^2^ cell a field plot based on the similarity of their BA, Dg and BA _b_. These 3 variables were chosen for matching because together they provide a synthetic yet fairly accurate picture of the stands.”** **Stem size heterogeneity or distribution would certainly bring another information layer. However, stem-related measurements (number, size, etc.) obtained from ALS are not very accurate. Including such variables in the procedure could in fact lead to less relevant matches.**  

 On page 6, what is meant by “Point cloud metrics were directly computed from the point cloud or(?) from the derived CHM”? I suggest to list all lidar metrics which were used in a table. **We removed the second part of the sentence “or from the derived CHM” for better clarity. As for the lidar metrics, see response to Reviewer 1’s comment on ALS metrics. ** 

 In Table 1, why are RMSE values for BAb > 1? In case they are given in percent, please add “(%)” to the caption. **RMSE values are indeed given in percent. We modified the table caption as suggested. ** 

 On page 9, the multiplication by 40000/pi and the division by 16 need to be explained. I suspect they convert values to the 1 ha and then back to the 25-m scale, however these scale factors should be explained explicitly. Also, the purpose of the rounding under “c)” should be better explained. **We added an explanation of the 40000 scale factor in a foot note as it appears in two different equations: “The scale factor 40000 is the product of two scale factors: 4 x 10000. The scale factor 4 comes from the formula linking a surface area *S* to a diameter *d* ( *S=π* *d* *4* *2*); while the scale factor 10000 accounts for the difference in units between the diameters (in cm) and the basal areas (in m ^2^).”**

**We also clarified the rounding procedure and the use of the scale factor 16 by modifying the “c)” paragraph as follows:   “c) Finally, we divided *ω* by 16 to get the weight of the trees in the 25×25 m ^2 ^cells ( *ω* being a weight per ha and 16 being the surface area ratio between 1 ha and a 25×25 m ^2^ cell). In doing so, the obtained tree weights can be either integer or decimal. However, the objective of our algorithm is to generate for each cell a list of individual trees with their associated diameter, height and species. From this perspective, decimal weights are not useful. We cannot simply round the tree weights to the nearest integer as this can lead to a significant over- or underestimation of the total number of trees in the cells. This is because the decimal part of the tree weights in the 25×25 m ^2^ cells is not the result of a random draw but directly depends on the surface area ratio between the field plot and the cell. As an example: 1 tree inventoried on a 400 m ^2^ field plot will always obtain a weight of 1.56 in a 25 x 25 m ^2^ cell, and a weight of 2 after rounding to the nearest integer. In order to obtain integer tree weights in the 25 x 25 m ^2^ cells while avoiding this bias, we performed a Bernoulli draw on the decimal part of the tree weights. As an example, a weight of 1.56 has a 56% chance of becoming 2, and a 44% chance of becoming 1. As this rounding of the weights slightly modifies the total BA of the generated stand, we transformed again the trees dbh to reach the total BA provided by the ALS mapping using the trees BA and their integer weights ( *ω _int_*) as follows: […]”**

 Figure 4: What is the explanation for the seemingly better fit (higher R²) in Milicz compared to Bauges? **The better predictions obtained at Milicz are discussed in the revised version of the validation section: “This higher quality of predictions can be explained by the fact that Milicz has the highest density of inventory plots and the least complex landscape, with a predominance of even-aged monospecific stands and the lowest species diversity among our three landscapes.” ** 

 General comments (for a possible Outlook): **We provide below some answers to the following two general comments. However, we would rather not include in the article the discussed points because our responses can only be speculative at this stage and further analyses would be required to provide solid answers. Our article is already rather dense for a data note, as pointed out by reviewer1, and we would prefer not to lengthen it further with speculative considerations. ** 

 Unlike an ITC approach, the presented method does not provide precise tree positions within the 25-m cells. Are there ways to expand the approach to additionally generate tree positions? **With the ABA and the LFI (local forest inventory) field plots as look up table, the tree lists in each pixel is very close to existing stands from the landscape. With an ITC method, the dominant trees and their position and heights can be retrieved, and the diameter and species estimated. In case the detection parametrization is chosen so as to avoid comission errors, stands need to be populated with additional trees to compensate for the omission errors. Our proposed workflow could be adapted to assign detected positions to trees in the list according to their sorted heights and then to randomly (or based on a model) assign positions to trees with no detected position.   Another possibility would be to use a semi-ITC approach as proposed by [https://doi.org/10.1016/j.rse.2009.12.004] to assign tree groups from detected clusters in the LFI reference plots to similar detected clusters in the landscape. This approach is interesting as it:**

- **provides the coordinates of the main detected trees**
- **directly provides a tree list at the lidar « detection cluster » scale, which can then be aggregated in larger areas (pixels, polygons)**

**The main drawback with this approach is that it requires a point density compatible with ITC analysis. In the southern part of the Bauges study area, the point density is too low to implement this approach.**

 Would it be possible/useful to add a height correction algorithm based on ALS heights (local maxima), similar to the dbh adjustment algorithm? **Our approach is based on the fact that stands basal area (BA), quadratic diameter (Dg) and density (N) are deterministically linked. As it stands, it seems difficult to add a height correction based on ALS heights to our approach. There would be one too many unknowns in the equation system, making it intractable.   A different approach could be developed, where instead of trying to reach the stands BA by modifying tree diameters, the aim would be to reach the stands total volume by modifying individual tree volumes. For this purpose, stand volume models from ALS point cloud should be created as well as models predicting individual tree volume as a function of tree height and diameter. The latter could be constrained by ALS heights.   However, volume allometries come with their own uncertainty and whether the generated stands would be more realistic following this procedure remains to be tested. More generally, there might be a trade-off between adjusting to the local lidar values, and making sure that we create an unbiased landscape with valid stands.**  

 Comments about the data:  

 The information about the coordinate reference system is missing. I was not able to georeference the asc files in a GIS. T **his is indeed an oversight. Thank you for pointing it out. We specified the coordinate reference systems for each site in the presentation of our dataset on the zenodo website and in the Extended data section:   “We also provide, for each virtual landscape, a raster (in .asc format) with the cell IDs (cellID25) which makes data spatialisation possible. The coordinate reference systems are EPSG: 2154 for the Bauges, EPSG: 2180 for Milicz, and EPSG: 3912 for Sneznik.”   We also added the coordinate reference systems in the R script provided alongside our dataset on the zenodo website. We took this opportunity to replace the raster package, which may no longer be fully maintained in the near future, with the terra package.**  

 It would be better to use unique file names, e.g. “milicz_cellID25.asc” etc. to be able to load all rasters in one GIS session. **Modified as suggested**

# Reviewer report: Reviewer response for version 1

**DOI:** 10.21956/openreseurope.16618.r30982

**Published:** 2023-04-04

**Authors:** Fabian Fischer

**Affiliations:** 1 School of Biological Sciences, University of Bristol, Bristol, England, UK

Overall assessment

 The article by Aussenac *et al.* describes a statistical procedure to generate a large data set of individual trees from airborne laser scanning (ALS) and inventory data. The variables include trunk diameter, tree height and species identity, and are provided across three European landscapes. The result is an impressive number of simulated/potential trees, which is a useful data set in forest ecology. As applications, the authors mention studies of scale and (more vaguely) forest management/ecosystem prediction, but one could easily think of a number of other concrete applications, such as input/validation of individual-based models of forest dynamics, or comparisons with automatically mapped tree crowns from airborne imagery, e.g. as in Weinstein *et al.* 2021 ^1^, Ball *et al*. 2022 ^2^, or spaceborne imagery, as in Tucker, Brandt, Hiernaux, *et al.* 2023 ^3^.

 I also found the paper generally well-written and with a well-thought through methodology for the mapping. The authors carefully tune their models to obtain optimal performance at every step and clearly have spent considerable amounts of time and effort to improve the prediction of stand attributes. In particular, I found the idea of matching predicted basal area to real stands and then filling in/removing trees until the basal area matches intriguing. This bears similarities with model-based estimations of forest attributes/tree attributes from lidar (Hurtt *et al.* 2004 ^4^, Taubert *et al*. 2015 ^5^, Rödig *et al.* 2017 ^6^, Fischer *et al*. 2020 ^7^) and shares some of these models’ advantages (e.g. more fine-scale distribution of biomass, no shrinking to the mean).

 However, like these models, the authors' method also involves a lot of complex modelling steps, and it is in the validation step of the procedure that I see deficiencies that need to be addressed. I see two main issues:

 a) the robustness of the models to extrapolation issues and spatial autocorrelation is not evaluated, so it is hard to assess how good the models are outside their calibration range and how much we can trust the predictions across the landscape.

 b) two of the key attributes of the data set (tree diameter and species identity) are not validated at all, despite featuring prominently in the title and in the results section (Figure 5). This should be a priority in a revised version. 

 In the following I will provide a few comments on the article following roughly the overall structure, and give suggestions on how to improve the model validation.

Justification for the data set

 I see the value of a fine-grain large-scale data set, and having such a data set is indeed rare, but it would be helpful to mention concrete applications. At the moment, the only justification given is the sentence: “Yet, this type of data could help address the scaling issues in ecology and could prove useful for testing forest management strategies and accurately predicting the dynamics of ecosystem services”. This is the sentence from the abstract, but the same point is made at the end of the first paragraph. Could the authors rephrase and add literature references in the main text? The vast majority of data sets can be useful for the testing of forest management strategies or predicting dynamics of ecosystem services. What is unique to your data set? Why do we need detailed, tree-based data at large scales?

Model for mapping of tree attributes

 ALS metrics: which metrics precisely did you use?

 Point cloud properties: Could the authors add information on/discussion of the sensitivity of their point metrics to scanner acquisitions? Lidar scans often exhibit considerable variation in pulse density even within a single acquisition (e.g. scan line centre vs. overlapping scan lines). What is each scan’s standard deviation of point/pulse density? Could you include that as a variable in stratification? Could this improve your models (e.g. stratify by pulse densities between 5 and 10, 10 and 15, 15 and 20, etc., or even smaller step sizes)?

 Descriptions: I appreciate that the paper is already quite dense, but quite a few steps in the methods section remain unclear to me, particularly in step 3. E.g., in the matching of BA and BAb, why do you need a correction value alpha? Can you explain the weighting better and why it is divided by 16? Maybe this is more exhaustively explained in the Extended Data, but this needs to be clear from the main text already.  

Model validation

 As pointed out above, this is the point of the paper that needs to be more comprehensive. At the moment, the authors validate their approach by comparing dominant height, as obtained from lidar (mean height of six highest local maxima), to dominant height of the simulated stands, obtained via local allometries (mean height of six highest trees). It is definitely useful to do this comparison and good to see that the results are broadly consistent, so I would keep it in the paper. However, there are issues with circularity, as the authors first use a number of lidar metrics that involve height / basal area-to-height relationships to create the maps and then compare the inferred results (+ independently derived height allometries) again to lidar-derived height metrics. Furthermore, height of the dominant trees may be related to basal area, but it cannot be used to evaluate basal area/tree diameter predictions as such, nor does it validate predicted species composition - both are key features of the data set.

 Given that the author’s simulation approach seems fast (only ca. 5 hours on a modern laptop, amazing!), another approach suggests itself, namely within-site cross-validation, ideally in the form proposed by Ploton *et al.* 2020 ^8^. Since a spatially explicit leave-one-out cross-validation, as suggested in Ploton *et al.* 2020 ^8^, may be too computationally intensive, I would recommend the simpler approach proposed in the same paper: for each of the European landscapes, I would recommend the authors to split their field data sets into, e.g., 5 spatially aggregated folds (i.e., spatial clusters), and run their model 5 times, each times using 4 folds to train the model and 1 separate geographic fold of plots to validate the model. In this 1 fold, the authors could directly compare predictions of tree values to actual data according to some simple standard metrics (total basal area, mean quadratic diameter, 95 ^th^ percentile of diameter, percentage of species xyz, 95 ^th^ percentile of height, mean height, dominant height). For comparison and to broadly assess whether spatial autocorrelation makes a difference, the authors could do the same validation procedure also with 5 folds containing plots randomly distributed in space (so no spatial clusters). This would only take 25 hours for each validation and give a good impression of how easy it is to accurately map individual trees and species at landscape scale and how realistic the produced inventories are. It would likely also increase interest in the data set, as it would give potential users higher confidence in the results.

 Since the paper puts its focus on the value of individual trees, there should, in my opinion, also be one result/validation graph that shows individual trees in some way. It could be, for example, a zoomed-in image of lidar-derived canopy height models + a predicted distribution of trees. If the 5-fold cross-validation is carried out, as above, the authors could simply show sample lidar canopy height models on top of plots, and the diameter distributions for the simulated and the inferred plots.

 Overall, it would also be interesting to readers to understand in how far the predicted species distributions reflect current expert knowledge, but this is not a necessity.

Data set

 I had a quick look at the data set. One variable I did not understand was the variable “n” or “number of trees”. Could you explain it a bit better? Does this mean that the specific diameter exists n times in the specific data set? If this is true (and only in this case), I seem to get some cells (very few) of 25m by 25m (e.g. cellID25 = 2439821 in the “Bauges” data set) that contain more than 500 trees with dbh >= 9-10cm per 625m2 and a total basal area >= 6m2 (which would yield roughly 100m ^2^ per hectare, at densities of 8000 trees). These are outliers, and every model is allowed to have outliers (and nature is full of them too), but it would be interesting to get your take on that in terms of realism/stand type. It could also be part of the validation to assess the edges of the basal area distribution or to give readers a hint what to make of the most extreme values.

Are sufficient details of methods and materials provided to allow replication by others?

Yes

Is the rationale for creating the dataset(s) clearly described?

Yes

Are the datasets clearly presented in a useable and accessible format?

Yes

Are the protocols appropriate and is the work technically sound?

Partly

Reviewer Expertise:

My areas of expertise are in lidar processing, individual-based modelling, as well as the creation of simulated forest stands (cf. my 2020 paper on this topic, mentioned in the review), which is very close to what the authors have been working on.

I confirm that I have read this submission and believe that I have an appropriate level of expertise to confirm that it is of an acceptable scientific standard, however I have significant reservations, as outlined above.

## References

[1] : A remote sensing derived data set of 100 million individual tree crowns for the National Ecological Observatory Network. *Elife* .2021;10: 10.7554/eLife.62922 10.7554/eLife.62922 33605211 PMC7895524

[2] : Accurate delineation of individual tree crowns in tropical forests from aerial RGB imagery using Mask R-CNN. *bioRxiv* .2022; 10.1101/2022.07.10.499480 10.1101/2022.07.10.499480

[3] : Sub-continental-scale carbon stocks of individual trees in African drylands. *Nature* .2023;615(7950) : 10.1038/s41586-022-05653-6 80-86 10.1038/s41586-022-05653-6 36859581 PMC9977681

[4] : Beyond Potential Vegetation: Combining Lidar Data and a Height-Structured Model for Carbon Studies. *Ecological Applications* .2004;14(3) : 10.1890/02-5317 873-883 10.1890/02-5317

[5] : The structure of tropical forests and sphere packings. *Proc Natl Acad Sci U S A* .2015;112(49) : 10.1073/pnas.1513417112 15125-9 10.1073/pnas.1513417112 26598678 PMC4679035

[6] : Spatial heterogeneity of biomass and forest structure of the Amazon rain forest: Linking remote sensing, forest modelling and field inventory. *Global Ecology and Biogeography* .2017;26(11) : 10.1111/geb.12639 1292-1302 10.1111/geb.12639

[7] : A simulation method to infer tree allometry and forest structure from airborne laser scanning and forest inventories. *Remote Sensing of Environment* .2020;251: 10.1016/j.rse.2020.112056 10.1016/j.rse.2020.112056

[8] : Spatial validation reveals poor predictive performance of large-scale ecological mapping models. *Nat Commun* .2020;11(1) : 10.1038/s41467-020-18321-y 4540 10.1038/s41467-020-18321-y 32917875 PMC7486894

# Response

**Published:** 2023-11-24

**Authors:** Raphaël Aussenac

**Affiliations:** CIRAD, France

**We would like to thank the reviewers for their useful comments which have helped significantly improve the manuscript. A special effort has been made to provide an in-depth evaluation of the generated landscapes, to clarify the functioning of our downscaling algorithm and to consolidate the rational of our work.**

**This revision has led us to optimise our code (better memory management, more effective parallelisation, etc.). It is now much faster, taking less than 1 hour to generate the 42 million trees of our three landscapes, while it used to take “less than 5 hours” to generate the 35 million trees of the Bauges landscape. We updated the introduction accordingly.**

**Please find below our replies to your comments.**

 The article by Aussenac *et al.* describes a statistical procedure to generate a large data set of individual trees from airborne laser scanning (ALS) and inventory data. The variables include trunk diameter, tree height and species identity, and are provided across three European landscapes. The result is an impressive number of simulated/potential trees, which is a useful data set in forest ecology. As applications, the authors mention studies of scale and (more vaguely) forest management/ecosystem prediction, but one could easily think of a number of other concrete applications, such as input/validation of individual-based models of forest dynamics, or comparisons with automatically mapped tree crowns from airborne imagery, e.g. as in Weinstein *et al.* 2021 ^1^ , Ball *et al*. 2022 ^2^ , or spaceborne imagery, as in Tucker, Brandt, Hiernaux, *et al.* 2023 ^3^ .

 I also found the paper generally well-written and with a well-thought through methodology for the mapping. The authors carefully tune their models to obtain optimal performance at every step and clearly have spent considerable amounts of time and effort to improve the prediction of stand attributes. In particular, I found the idea of matching predicted basal area to real stands and then filling in/removing trees until the basal area matches intriguing. This bears similarities with model-based estimations of forest attributes/tree attributes from lidar (Hurtt *et al.* 2004 ^4^ , Taubert *et al*. 2015 ^5^ , Rödig *et al.* 2017 ^6^ , Fischer *et al*. 2020 ^7^ ) and shares some of these models’ advantages (e.g. more fine-scale distribution of biomass, no shrinking to the mean).

 However, like these models, the authors' method also involves a lot of complex modelling steps, and it is in the validation step of the procedure that I see deficiencies that need to be addressed. I see two main issues:

 a) the robustness of the models to extrapolation issues and spatial autocorrelation is not evaluated, so it is hard to assess how good the models are outside their calibration range and how much we can trust the predictions across the landscape. **The leave-one-out cross validation (LOOCV) performed on our entire workflow presented in this revised version of the article gives an indication on the robustness of our approach (see responses to the comments on model validation).** 

**Regarding lidar modelling, cross-validation (such as used in this study) remains a common method for accuracy assessment of landscape predictions, although some limitations have been highlighted in the literature. Accuracy assessment remains an important research topic, with no consensus on the best practices, as field data are usually scarce and modeling approaches complex (see paragraph 2.5 of the review in https://doi.org/10.1016/j.rse.2021.112477).**

**We acknowledge that spatial autocorrelation is necessarily present both in the lidar acquisition and in the forest structure. Hence spatial autocorrelation can be expected in the lidar model predictions and errors. Unfortunately, calibration plots were sampled on a regularly spaced grid in each study area which does not make it possible to quantify spatial autocorrelation at distances smaller than the grid step.**

 b) two of the key attributes of the data set (tree diameter and species identity) are not validated at all, despite featuring prominently in the title and in the results section (Figure 5). This should be a priority in a revised version. **In this revised version, we provide an in-depth evaluation of the generated landscapes (see responses to the specific comments below).   We changed the abstract accordingly: “We carried out an in-depth evaluation of our workflow including, among other analyses, a leave-one-out cross validation. Overall, the landscapes we generated are in good agreement with the landscapes they aim to reproduce. In the most favourable conditions, the root mean square error (RMSE) of stand basal area (BA) and mean quadratic diameter (Dg) predictions were respectively 5.4 m ^2^ and 3.9 cm, and the generated main species corresponded to the observed main species in 76.2% of cases.”**

**We also modified the paragraph presenting the evaluation in the General Approach section: “We evaluated the overall reliability of our workflow, i.e. its ability to produce virtual landscapes as close as possible to the real ones (see Dataset validation). In particular we carried out a leave-one-out cross validation (LOOCV) on our entire workflow. This analysis consisted in: • comparing the observed and predicted values of BA, Dg, Bab and the quantiles of tree height and diameter; • comparing the observed and predicted values of species abundance at the landscape level; • calculating the frequency at which the most abundant species was correctly predicted at the cell level; As a complement, we also compared the stands dominant heights measured by ALS (HdomALS) to those calculated from the trees we generated (HdomT). Finally, we compared the spatial distribution of species to current expert knowledge.”**

 In the following I will provide a few comments on the article following roughly the overall structure, and give suggestions on how to improve the model validation.

Justification for the data set

 I see the value of a fine-grain large-scale data set, and having such a data set is indeed rare, but it would be helpful to mention concrete applications. At the moment, the only justification given is the sentence: “Yet, this type of data could help address the scaling issues in ecology and could prove useful for testing forest management strategies and accurately predicting the dynamics of ecosystem services”. This is the sentence from the abstract, but the same point is made at the end of the first paragraph. Could the authors rephrase and add literature references in the main text? The vast majority of data sets can be useful for the testing of forest management strategies or predicting dynamics of ecosystem services. What is unique to your data set? Why do we need detailed, tree-based data at large scales? **As suggested, we rephrased this part and added some references:  “Yet, fine-grain descriptions of large forest areas could help address the pervasive scaling issues in forest ecology, modelling and management. In practice, such data could help better understand at which spatial scale ecological processes emerge in forest ecosystems [Craven et al., 2020; With, 2019]. They could also be extremely valuable to compare forest dynamics models operating at different scales (organ, tree, stand, landscapes) and evaluate their validity across scales [Papaik et al., 2010]. They could ultimately help develop and test management strategies at different spatial scales [Seidl et al., 2013].”   Added references: Craven, Dylan, et al. "A cross‐scale assessment of productivity–diversity relationships." Global Ecology and Biogeography 29.11 (2020): 1940-1955. Papaik, Michael J., et al. "Forest processes from stands to landscapes: exploring model forecast uncertainties using cross-scale model comparison." Canadian journal of forest research 40.12 (2010): 2345-2359. Seidl, Rupert, et al. "deepl" European Journal of Forest Research 132 (2013): 653-666.**

Model for mapping of tree attributes

 ALS metrics: which metrics precisely did you use? **Candidate metrics for selection are those computed with the aba_metrics and std_tree_metrics functions from the lidaRtRee R package, as stated in section ALS mapping. Selected metrics result from an automated procedure applied separately on each study area, forest parameter, stratum (in case a specific model is calibrated for each stratum). The following sentence was added to the Stratification”subsection of the ALS mapping section: “The metrics selected in the 32 models for BA and Dg (which include at most six independent variables) are presented in Table S1 of the *Extended data*.”**

**The seven stratum-specific logistic regression models obtained for BA _b_ with stepaAIC variable selection include 20 to 30 metrics and are not presented.**

 Point cloud properties: Could the authors add information on/discussion of the sensitivity of their point metrics to scanner acquisitions? **Visual inspection of metrics maps was performed to exclude metrics exhibiting spatial patterns which might be linked to acquisition. We hypothesise that the selected metrics and models based on them are robust to variations in acquisition settings, as a systematic sampling of field plots should ensure that the heterogeneities of lidar acquisition and forest structure on the whole landscape are represented in the data. In the case of Milicz, several metrics seemed quite sensitive to points acquired with a large scan angle. Scan angle values above 21 degrees were excluded in order to achieve a trade-off between metrics robustness, point density and comprehensive coverage of the study area. In the case of Sneznik, some intensity-related metrics where also found to be influenced by acquisition pattern and where thus removed from the analysis ("imean", "imax", "isd", "iskew", "ikurt"). The following sentences were added to the manuscript: “Each metric map was visually checked for spatial patterns potentially linked to acquisition patterns, which eventually led to: - discard some intensity-related metrics in Sneznik study area; - remove ALS points acquired with a scan angle larger than 21 degrees in Milicz study area, in order to achieve a trade-off between metrics robustness, point density and comprehensive coverage of the study area.” ** 

 Lidar scans often exhibit considerable variation in pulse density even within a single acquisition (e.g. scan line centre vs. overlapping scan lines). What is each scan’s standard deviation of point/pulse density? **The mean +- sd point density (/m2) for each study site, computed at 25 m resolution for pixels with at least 1 point above 2 m height is : Bauges (southern part) : 5.9 +- 3.1 Bauges (northern part) : 27.6 +- 13.3 Milicz : 16.5 +- 7.1 Sneznik : 18.4 +- 10.1 Those values are now indicated in the article. Mean values slightly differ from the previous ones which were computed for pixels of the whole landscapes.**  

 Could you include that as a variable in stratification? Could this improve your models (e.g. stratify by pulse densities between 5 and 10, 10 and 15, 15 and 20, etc., or even smaller step sizes)? **In order to obtain robust models and valid inferences, [https://doi.org/10.5589/m12-052] suggests to use at least 55 observations in calibration, which limits the number of strata that the whole dataset can be partitioned in. We hypothesised that a stratification based on forest structure defined by ancillary data would help in reducing the prediction error. For the Bauges study area where ALS data originate from two different ALS campaigns, campaign was also tested as stratification criterion. Several stratifications where tested, and the one retained for the final map production is the one with the best RMSE improvement in cross validation compared to the single model. Parcimony was also considered when selecting the model. We have not tested a stratification based on pulse densities, as most computed metrics are a priori robust to density and as we have checked that metrics maps did not display any pattern due to acquisition conditions, such as density variations.**

 Descriptions: I appreciate that the paper is already quite dense, but quite a few steps in the methods section remain unclear to me, particularly in step 3. E.g., in the matching of BA and BAb, why do you need a correction value alpha? Can you explain the weighting better and why it is divided by 16? Maybe this is more exhaustively explained in the Extended Data, but this needs to be clear from the main text already. **We have gone into more detail on points 3a and 3b. They now read as follows:**

-
  - **“(a) For this, we first calculated *α*, a multiplier correction coefficient to be applied to all tree diameters of a field plot. The idea is to increase or decrease tree diameters so that their Dg reaches the Dg value of the cell to which they are associated. *α* is given by:”**
  - **“(b) Thereafter, we calculated the weight (ω in n.ha ^-1^) of these trees with corrected diameters, so that the generated stand matches the BA and BA _b_ values of the cell to which it is associated. ω is given by:”**

**As for the weighting, it is now explained (in a foot note and in point 3c), but see answer to Reviewer 2’s comment on scale factors.**

Model validation

 As pointed out above, this is the point of the paper that needs to be more comprehensive. At the moment, the authors validate their approach by comparing dominant height, as obtained from lidar (mean height of six highest local maxima), to dominant height of the simulated stands, obtained via local allometries (mean height of six highest trees). It is definitely useful to do this comparison and good to see that the results are broadly consistent, so I would keep it in the paper. However, there are issues with circularity, as the authors first use a number of lidar metrics that involve height / basal area-to-height relationships to create the maps and then compare the inferred results (+ independently derived height allometries) again to lidar-derived height metrics. Furthermore, height of the dominant trees may be related to basal area, but it cannot be used to evaluate basal area/tree diameter predictions as such, nor does it validate predicted species composition - both are key features of the data set. **We agree with the reviewer, the comparison of Hdom only partially validates our virtual landscapes. The revised version of the article includes a much more comprehensive evaluation of the virtual landscapes.**

**We added this point on circularity in the section presenting the comparation of Hdom as follows: “There is some circularity in comparing Hdom ALS and Hdom T as models predicting BA, Dg and BA b from ALS point clouds may include ALS derived height metrics or more generally metrics which are correlated with the dominant height estimated from ALS point clouds. The results of this comparison must therefore be interpreted with caution.”**

 Given that the author’s simulation approach seems fast (only ca. 5 hours on a modern laptop, amazing!), another approach suggests itself, namely within-site cross-validation, ideally in the form proposed by Ploton *et al.* 2020 ^8^ . Since a spatially explicit leave-one-out cross-validation, as suggested in Ploton *et al.* 2020 ^8^ , may be too computationally intensive, I would recommend the simpler approach proposed in the same paper: for each of the European landscapes, I would recommend the authors to split their field data sets into, e.g., 5 spatially aggregated folds (i.e., spatial clusters), and run their model 5 times, each times using 4 folds to train the model and 1 separate geographic fold of plots to validate the model. In this 1 fold, the authors could directly compare predictions of tree values to actual data according to some simple standard metrics (total basal area, mean quadratic diameter, 95 ^th^ percentile of diameter, percentage of species xyz, 95 ^th^ percentile of height, mean height, dominant height). For comparison and to broadly assess whether spatial autocorrelation makes a difference, the authors could do the same validation procedure also with 5 folds containing plots randomly distributed in space (so no spatial clusters). This would only take 25 hours for each validation and give a good impression of how easy it is to accurately map individual trees and species at landscape scale and how realistic the produced inventories are. It would likely also increase interest in the data set, as it would give potential users higher confidence in the results. **We carried out a leave-one-out cross-validation (LOOCV). This validation is actually not too computationally intensive because it has to run only on a few hundred field plots and not on the whole landscape. We also preferred this LOOCV to a k-fold cross-validation because the latter would require to check that sample size in each stratum would remain relevant in each calibration fold, while performing repetitions of the k-fold samples to assess the reliability of the statistics. Besides we are not certain that spatially correlated folds will help in understanding the robustness of the map. One can expect a model calibrated on a spatially-selected subset to perform poorly on another spatial subset as the predicted area may be different from the calibration area both for the lidar and forest. Spatial k-folds do not allow to assess the local spatial variations of performance of a model calibrated with systematic samples (which is what our models are) in a better way than what a LOOCV does. In both cases, spatial correlation at distances smaller than the grid step, which is interesting e.g. for small area estimations, is impossible to evaluate.**  

**We added the following paragraph to the method subsection of the validation section:   “We carried out a leave-one-out cross validation (LOOCV) to evaluate the realism of the virtual landscapes we generated. This consisted in excluding a field plot from our entire workflow and comparing the predicted values obtained to the observed values. This operation was repeated within each landscape for all field plots. We calculated the root mean square error (RMSE) of the predictions of BA, Dg, Bab and the quantiles of tree height and diameter. As part of the LOOCV, we also compared the observed and predicted values of species abundance at the landscape level (in BA) and calculated the frequency at which the most abundant species was correctly predicted at the cell level.”**

**We presented the results of this new evaluation procedure in the results subsection of the validation section: “Overall, the virtual landscapes are in good agreement with the landscapes they aim to reproduce. The generated stand structures and compositions are consistent with the observations and make it possible to distinguish stands at different stages of development and with different compositions.**

**At Milicz, predictions are the most accurate. The RMSE of all evaluated variables are the lowest in comparison with the other landscapes (Table T1, Figure 4). Species abundance at the landscape level is also better reproduced (Figure F1). Finally, in 76.2% of cases, the generated main species corresponds to the observed main species. This higher quality of predictions can be explained by the fact that Milicz has the highest density of inventory plots and the least complex landscape, with a predominance of even-aged monospecific stands and the lowest species diversity among our three landscapes.**

**At the Bauges and Sneznik, the RMSE of the evaluated variables are comparable (Table T1., Figure 4). In contrast, predictions of species abundance at the landscape level are more accurate at Sneznik (Figure F1). The same applies to the compositions predicted at the plot level: the predicted main species corresponds to the observed main species in 63.1% of cases at Sneznik and in 37.2% of cases at the Bauges. However, two datasets were used in the Bauges. In the local forest inventory (LFI) not all trees were identified at the species level and trees with a dbh between 7.5 and 17.5 cm were not measured but counted by diameter classes and grouped in two categories (coniferous and broadleaf). This led us to use a local subset of the NFI from which composition is derived in our downscaling algorithm. The poorer composition predictions in the Bauges might therefore partly be an artefact arising from the evaluation itself, as the LFI may not be suitable to serve as a field reference.**

**The fact that the RMSE values obtained from the LOOCV carried out on our entire workflow are almost similar to the RMSE values obtained from the LOOCV of ALS models shows that the downscaling algorithm hardly adds any error (Table 1, Table T1). The main way of increasing the realism of our virtual landscapes would therefore be to improve the ALS models.”**

**Finally, we added the RMSE values associated to the three scatter plots of our Figure 4 for greater consistency in the document and modified the paragraph where this figure is presented: “With R ^2^ values ranging from 0.61 to 0.83 (Figure 4) and RMSE values below 5 m, Hdom _ALS_ and Hdom _T_ are consistent with one another. This provides a general validation of our workflow. As discussed above, the better predictions obtained at Milicz might steam from the higher density of inventory plots and the lower complexity of the landscape. At Sneznik, Hdom _T_ tends to be overestimated as Hdom _ALS_ decreases. This divergence could be due to the ice storm that occurred between the field inventory and the ALS acquisition and that might have biased the ALS models.”**

 Since the paper puts its focus on the value of individual trees, there should, in my opinion, also be one result/validation graph that shows individual trees in some way. It could be, for example, a zoomed-in image of lidar-derived canopy height models + a predicted distribution of trees. If the 5-fold cross-validation is carried out, as above, the authors could simply show sample lidar canopy height models on top of plots, and the diameter distributions for the simulated and the inferred plots. **Here, we do not exactly agree with the reviewer. We do not generate individual trees, per se, but rather a list of trees for each cell, and those generated trees are not spatialised within the cells. For such a purpose different methods and denser ALS data would be required. Also, it is not clear to us how the comparison of canopy height models and diameter distributions would constitute an evaluation of our algorithm. The comparison of diameter and height quantiles carried out in response to the previous comment seems to us a better way to evaluate the quality of the generated tree lists.**

 Overall, it would also be interesting to readers to understand in how far the predicted species distributions reflect current expert knowledge, but this is not a necessity. **In the method subsection of the validation section, we added: “Finally, we examined the spatial distribution of species at each site and compared it to current expert knowledge.”**

**And in the results subsection of the validation section, we added: “Overall, species spatial distribution in the virtual landscapes is consistent with field observations. In the Bauges, pure and mixed stands of fir and spruce are more abundant at higher elevation while mixed stands of broadleaf species are found at lower elevation. At Milicz, pure stands of Scots pine are found at lower elevation while broadleaf species and mixed stands appear at higher elevation. Finally, at Sneznik, pure beech stands are found at higher elevation while fir is found at lower elevation in pure or mixed stands (a specific feature of the site).”**

Data set

 I had a quick look at the data set. One variable I did not understand was the variable “n” or “number of trees”. Could you explain it a bit better? Does this mean that the specific diameter exists n times in the specific data set? If this is true (and only in this case), I seem to get some cells (very few) of 25m by 25m (e.g. cellID25 = 2439821 in the “Bauges” data set) that contain more than 500 trees with dbh >= 9-10cm per 625m2 and a total basal area >= 6m2 (which would yield roughly 100m ^2^ per hectare, at densities of 8000 trees). These are outliers, and every model is allowed to have outliers (and nature is full of them too), but it would be interesting to get your take on that in terms of realism/stand type. It could also be part of the validation to assess the edges of the basal area distribution or to give readers a hint what to make of the most extreme values. **The variable “n” indeed means that a specific diameter exists n times. We clarified that in the presentation of our dataset on the zenodo website and in the Extended data section as follows: “- n: number of trees. n is an integer >= 1, meaning that a specific set of species “sp”, diameter “dbh” and height “h” can be present multiple times in a cell.”**

**The extreme values you identified are indeed outliers. They are a direct consequence of the uncertainties associated with the models. The realism of the stands associated with these extreme values is, of course, open to question. However, it seems difficult to define thresholds separating realistic from unrealistic stands as extreme values can be locally observed. We believe it is up to the users of the dataset to decide whether or not to use all the stands depending on their objectives. We drew the reader’s attention to the outliers at the end of the validation section as follows: “Our procedure is not free of flaws and some outliers are present in the generated data (i.e. stands with extreme values of BA, Dg, tree height or density). These outliers are a direct consequence of the uncertainties associated with the models we used. The realism of the stands associated with these extreme values is open to question. However, separating realistic from unrealistic stands seems difficult as extreme values can be locally observed. It is therefore up to the users of the dataset to decide whether or not to consider these stands depending on their objectives.”**

**Finally, it is always tricky to assess the relevance of extreme values as the field data itself does not allow to estimate them accurately. We believe such an analysis could hardly help in evaluating our approach and we would rather not include it as part of the validation.**
